# miR-29a-3p in Exosomes from Heme Oxygenase-1 Modified Bone Marrow Mesenchymal Stem Cells Alleviates Steatotic Liver Ischemia-Reperfusion Injury in Rats by Suppressing Ferroptosis via Iron Responsive Element Binding Protein 2

**DOI:** 10.1155/2022/6520789

**Published:** 2022-06-09

**Authors:** Xiang Li, Longlong Wu, Xuan Tian, Weiping Zheng, Mengshu Yuan, Xiaorong Tian, Huaiwen Zuo, Hongli Song, Zhongyang Shen

**Affiliations:** ^1^Tianjin First Central Hospital Clinic Institute, Tianjin Medical University, Tianjin 300070, China; ^2^School of Medicine, Nankai University, Tianjin, China; ^3^Department of Organ Transplantation, Tianjin First Central Hospital, Tianjin 300192, China; ^4^NHC Key Laboratory of Critical Care Medicine, Tianjin 300192, China; ^5^Tianjin Key Laboratory of Organ Transplantation, Tianjin, China; ^6^Key Laboratory of Transplant Medicine, Chinese Academy of Medical Sciences, Tianjin, China

## Abstract

Hepatic ischemia-reperfusion injury (IRI) is an inevitable result of liver surgery. Steatotic livers are extremely sensitive to IRI and have worse tolerance. Ferroptosis is considered to be one of the main factors of organ IRI. This study is aimed at exploring the role of ferroptosis in the effect of heme oxygenase-1-modified bone marrow mesenchymal stem cells (HO-1/BMMSCs) on steatotic liver IRI and its mechanism. An IRI model of a steatotic liver and a hypoxia reoxygenation (HR) model of steatotic hepatocytes (SHPs) were established. Rat BMMSCs were extracted and transfected with the *Ho1* gene to establish HO-1/BMMSCs, and their exosomes were extracted by ultracentrifugation. *Ireb2* was knocked down to verify its role in ferroptosis and cell injury in SHP-HR. Public database screening combined with quantitative real-time reverse transcription PCR identified microRNAs (miRNAs) targeting *Ireb2* in HO-1/BMMSCs exosomes. miR-29a-3p mimic and inhibitor were used for functional verification experiments. Liver function, histopathology, terminal deoxynulceotidyl transferase nick-end-labeling staining, cell viability, mitochondrial membrane potential, and cell death were measured to evaluate liver tissue and hepatocyte injury. Ferroptosis was assessed by detecting the levels of IREB2, Fe^2+^, malondialdehyde, glutathione, lipid reactive oxygen species, glutathione peroxidase 4, prostaglandin-endoperoxide synthase 2 mRNA, and mitochondrial morphology. The results revealed that HO-1/BMMSCs improved liver tissue and hepatocyte injury and suppressed ferroptosis *in vivo* and *in vitro*. The expression of IREB2 was increased in steatotic liver IRI and SHP-HR. Knocking down *Ireb2* reduced the level of Fe^2+^ and inhibited ferroptosis. HO-1/BMMSC exosomes reduced the expression of IREB2 and inhibited ferroptosis and cell damage. Furthermore, we confirmed high levels of miR-29a-3p in HO-1/BMMSCs exosomes. Overexpression of miR-29a-3p downregulated the expression of *Ireb2* and inhibited ferroptosis. Downregulation of miR-29a-3p blocked the protective effect of HO-1/BMMSC exosomes on SHP-HR cell injury. In conclusion, ferroptosis plays an important role in HO-1/BMMSC-mediated alleviation of steatotic liver IRI. HO-1/BMMSCs could suppress ferroptosis by targeting *Ireb2* via the exosomal transfer of miR-29a-3p.

## 1. Introduction

With the increasing demand for liver transplantation, the shortage of organs has led to the application of expanded criteria donor (ECD) livers [1]. As the most common chronic liver disease in the world, a steatotic liver is a common type of ECD liver. However, severe hepatic steatosis is extremely sensitive to ischemia-reperfusion injury (IRI) and has low tolerance, which increases the incidence of serious complications, such as early graft dysfunction, primary graft nonfunction, and ischemic biliary disease after liver transplantation [2]. It is estimated that 25% of discarded donor livers result from severe steatosis of the liver [3]. Therefore, the problem of steatotic liver IRI needs to be solved urgently.

Bone marrow mesenchymal stem cells (BMMSCs) have the characteristics of easy access, a high proliferation rate, and low immunogenicity. They play an important role in organ injury repair and transplantation immune regulation [4–6]. However, the complex pathophysiological environment in the lesion, such as oxidative stress, seriously limits the survival of BMMSCs and reduces the efficacy of BMMSCs transplantation. Heme oxygenase-1 (HO-1) is a recognized antioxidant enzyme, which can protect cells from oxidative stress-related damage. Our previous study showed that HO-1 modification of BMMSCs (HO-1/BMMSCs) prolonged the survival time of BMMSCs in a complex pathological environment *in vivo*, optimized their activity, and efficacy, and better protected against IRI of liver transplantation in rats [7].

Exosomes have been proven to be important paracrine mediators and represent the mechanism of action of BMMSCs [8, 9]. At the same time, HO-1/BMMSCs changed the transcriptome expression profile of BMMSCs and the genetic profile of their exosomes, optimized the paracrine function of BMMSCs, and then enhanced and enriched their physiological regulation function [10]. Therefore, it would be interesting to further explore the protective effect of HO-1/BMMSCs on steatotic liver IRI and to determine their mechanism.

Ferroptosis is an iron-dependent pathway of inducing lethal lipid peroxidation, resulting in cell death [11]. Studies have shown that ferroptosis is related to a variety of human diseases, and inhibition of ferroptosis can reduce the occurrence and development of a steatotic liver and liver IRI [12–14]. In addition, the increase in liver iron content is an important pathogenic factor for the occurrence of a steatotic liver, and abnormal expression of iron metabolism-related proteins occurs in steatotic livers [15, 16]. At the same time, the increase of Fe^2+^ caused by the abnormal expression of iron metabolism-related proteins and the production of large amounts of reactive oxygen species (ROS) are also important factors for liver IRI [17]. Therefore, the severe liver injury caused by steatotic liver IRI might be related to the abnormal expression of iron metabolism-related proteins and the occurrence of ferroptosis.

Iron response element-binding protein 2 (IREB2) is the main regulator of cellular iron homeostasis. It regulates the translation of *FTH1* mRNA (encoding ferritin H, which mediates the storage of intracellular iron) and the stability of *TFR1* mRNA (encoding transferrin receptor 1, which mediates cellular iron transport) through posttranscriptional regulatory mechanisms and plays a central role in cellular iron homeostasis [18, 19]. *IREB2* has been proven to be a marker gene for ferroptosis [11, 20]. However, the effects of *IREB2* on ferroptosis of steatotic liver IRI are unclear.

In the present study, we explored the protective effect of HO-1/BMMSCs on steatotic liver IRI, steatotic hepatocyte (SHP) hypoxia and reoxygenation (HR), and its relationship with ferroptosis, including its specific regulation mechanism.

## 2. Materials and Methods

### 2.1. Animals

Clean-grade male Sprague-Dawley (SD) rats were purchased from China Food and Drug Administration (Beijing, China), with certificate number SCXK (Jing) 2017-0005. Four-week-old rats (weighing about 50 g) were used to isolate BMMSCs, and 8-week-old rats (about 220 g) were used to create the steatotic liver model. The rats were housed in a temperature- and humidity-controlled environment under a standard 12 h light/dark cycle with free access to food and water. All animal experiments were conducted in accordance with the ARRIVE guidelines 2.0: updated guidelines for reporting animal research and were approved by the Animal Ethics Committee of Nankai University (batch number: 2021-SYDWLL-000331).

### 2.2. Isolation and Identification of HO-1/BMMSCs

BMMSCs were isolated from rat femoral and tibial bone marrow, cultured using the continuous adherence method, and then transfected adenovirus expressing the *Ho1* gene (Genechem, Shanghai, China) to establish HO-1/BMMSCs [10]. Lipogenic and osteogenic differentiations were used to identify the differentiation potential of HO-1/BMMSCs. Antibodies against cluster of differentiation (CD)29, CD34, CD45, CD90, RT1 class I, locus A (RT1A), RT1 class I, and locus B (RT1B) (BioLegend, San Diego, CA, USA) were detected for the HO-1/BMMSCs phenotype using flow cytometry. *Ho1* expression was determined using quantitative real-time reverse transcription PCR (qRT-PCR), western blotting, and immunofluorescence staining.

### 2.3. Isolation, Identification, and Uptake of Exosomes

After 72h of HO-1/BMMSC culture in exosome-free serum medium, debris and dead cells in the medium were removed by centrifugation at 250×*g* for 15min and 3000×*g* for 30min and then filtered through a 0.22*μ*m filter. The medium was then subjected to ultracentrifugation at 110,000×*g* for 140min at 4°C. Exosomes were resuspended in sterile phosphate-buffered saline (PBS).

Exosome morphology was observed using a transmission electron microscope (Hitachi-HT7700, Tokyo, Japan). The exosomal particle size was determined using nanoparticle tracking analysis (Malvern-NS300, Malvern, UK). The levels of exosome-specific proteins CD9, CD63, tumor susceptibility 101 (TSG101), and calnexin (negative control; ProteinTech, Wuhan, China) were detected using western blotting.

To monitor exosome trafficking, exosomes were labeled with chloromethyl-1,1′-dioctadecyl-3,3,3′3′-tetramethylindocarbocyanine perchlorate (CM-DiI fluorescent dye, Invitrogen, Waltham, MA, USA) for 15 min in the dark at 4°C. After CM-DiI staining, the exosomes were washed in PBS and collected by ultracentrifugation (110,000×*g* for 70min) at 4°C. Finally, CM-DiI-labeled exosomes were resuspended in PBS. The rat liver cell line IAR20 was cocultured with the labeled exosomes for 6 h, and the uptake of exosomes was observed using a confocal microscope (FluoView™-FV1000, Olympus, Tokyo, Japan).

### 2.4. Steatotic Liver IRI Model and Treatment

SD rats were fed a high-fat diet (Composition: 15% triglyceride, 15% sucrose, 10% egg yolk powder, 1% cholesterol, 0.2% bile salt, 58.8% basic feed) for 20 weeks. Hematoxylin and eosin (HE) and oil red O staining showed that the area of mixed macrovesicular steatosis was more than 60% under the microscope, indicating that a model of severe steatotic liver was established successfully [21]. A 70% liver thermal ischemia model was established, continuously blocked for 80 min, and then, the ischemic liver was obtained 24 h after reperfusion [22]. According to different treatments, animals were randomly divided into the following groups: the sham operation group (Sham), the IRI group, the IRI treated with ferrostatin-1 (Fer-1, MCE, Monmouth Junction, NJ, USA) (IRI + Fer − 1) group, the IRI treated with BMMSCs (IRI + BM) group, and the IRI treated with HO-1/BMMSC (IRI + HBM) group. Rats were infused with 2 × 10^6^ BMMSCs or HO-1/BMMSCs 1 day before surgery (via the tail vein) and immediately after reperfusion (via the portal vein). Rats were given intraperitoneal Fer-1 treatment 1 day before surgery and immediately after reperfusion (5 mg/kg) in the IRI + Fer − 1 group. Each group comprised six rats.

### 2.5. HO-1/BMMSC Tracking

BMMSCs were transfected with adenovirus expressing the green fluorescent protein (GFP) (Genechem) or expressing both Ho-1 and GFP. An *in vivo* imaging system (Cambridge Research & Instrumentation, Inc. (CRi), Woburn, MA, USA) was used to observe the level of green fluorescence in the liver after 24 h of reperfusion. The liver was excised and made into frozen sections, and the colonization of BMMSCs and HO-1/BMMSCs in liver tissues was observed under a fluorescence microscope (IX71, Olympus).

### 2.6. Steatotic Hepatocyte (SHPs) Hypoxia and Reoxygenation (HR) Model and Treatment

IAR20 cells were seeded in 12-well plates and incubated with 20 *μ*L of oleate and 5 *μ*L of palmitate (Sigma-Aldrich, St. Louis, MO, USA) for 24 h to induce steatosis, which was verified using oil red staining. The HR model was established according to previously published methods [23, 24]. IAR20 cells were cultured in serum-free minimal essential medium (MEM) (Gibco, Thermo Scientific, Waltham, MA, USA) for 24 h. After washing with PBS, the cells were completely immersed in mineral oil (Aladdin, Shanghai, China) to cause hypoxia and placed in a 37°C, 5% CO_2_ incubator. After 4 h, the mineral oil was discarded and the cells were washed with PBS several times, and then, the hypoxic IAR20 cells were cultured in MEM containing 10% fetal bovine serum (FBS, Biowest, Nuaillé, France) to reoxygenate for 6 h. According to different treatments, the following groups were set: Sham, HR, HR treated with Fer-1 (1 *μ*M), HR treated with Emricasan (30 *μ*M) (MCE, Monmouth Junction, NJ, USA), HR treated with BM, HR treated with HBM, and HR treated with HO-1/BMMSCs exosomes (HBM-exo) groups. A coculture system of BM/HBM (1 × 10^5^ cells/well) with SHPs at a ratio of 1 : 1 was established using a 0.8 *μ*m Transwell chamber (Corning Inc., Corning, NY, USA) after reoxygenation. For HO-1/BMMSC experiments requiring exosomal inhibition *in vitro*, HO-1/BMMSCs cultured to 70–80% confluence were treated with GW4869 (MCE, Monmouth Junction, NJ, USA) at 20 *μ*M for 24 h before SHP-HR in a Transwell chamber. To clarify the role of HBM-exos, exosomes derived from HO-1/BMMSCs were added to the culture supernatant in the SHP-HR model at 1.5 × 10^9^ exosomes/mL.

### 2.7. siRNA, miRNA Mimic, and miRNA Inhibitor Transfection

Small interfering RNA (siRNA), an microRNA (miRNA) mimic, and an miRNA inhibitor were created and synthesized by GenePharma (Shanghai, China), with the sequences shown in Table S1 and Table S2. Cells were cultured in 6-well plates to achieve a confluence of 60% to 80% within 24 h. Transfection was performed using Lipofectamine™ 3000 (L3000015, Invitrogen, Carlsbad, CA, USA) according to the manufacturer's protocol. The miRNA mimic or siRNA was transfected into SHPs, and the miRNA inhibitor was transfected into HO-1/BMMSCs. After 24 h, the transfection efficiencies were validated using either qRT-PCR or western blotting. The HR model was created 48 h after transfection.

### 2.8. HE and TUNEL Staining

Liver tissues were fixed completely using 10% formalin solution, embedded in paraffin, and serially sectioned (3 *μ*m). HE and terminal deoxynucleotidyl transferase nick-end-labeling (TUNEL) staining were performed to comprehensively reflect liver pathological damage and liver cell death [25]. We randomly selected five microscope fields (at ×200 magnification) and observed and evaluated the Suzuki score [26] and the TUNEL positive cell percentage in different groups.

### 2.9. Liver Function

Serum alanine aminotransferase (ALT) and aspartate aminotransferase (AST) levels were measured using a fully automatic biochemical analyzer (Cobas 800; Roche Diagnostics, Basel, Switzerland).

### 2.10. Immunohistochemical Staining

Paraffin-embedded sections of liver tissue were subjected to immunohistochemical staining as described previously [27]. Primary antibodies recognizing 4-hydroxynonenal (4-HNE; 1 : 50, Abcam Inc., Cambridge, UK), CD68 (1 : 200, Santa Cruz Biotechnology, Dallas, TX, USA), myeloperoxidase (MPO) (1 : 200, ProteinTech), and IREB2 (1: 200, Santa Cruz Biotechnology) were incubated overnight at 4°C, followed by incubation with the secondary antibody and horseradish peroxidase-labeled streptavidin (HRP). Finally, after diaminobenzidine (DAB) staining and hematoxylin staining of the nucleus, the expression ratio of positive cells was determined under an optical microscope.

### 2.11. Measurement of Fe^2+^, Malondialdehyde (MDA), and Glutathione (GSH)

According to the manufacturer's instructions of the iron assay kit (Abcam), the MDA detection kit (Beyotime, Shanghai, China), and the GSH detection kit (Solarbio, Beijing, China), the absorbance of the supernatant after liver tissue homogenization was detected at wavelengths of 593 nm (Fe^2+^), 532 nm (MDA), and 412 nm (GSH), and then, the absorbance value was used to calculate the content of three indexes in terms of unit weight of liver tissue.

### 2.12. Transmission Electron Microscopy (TEM)

Fresh liver tissue was cut into 1 × 1 × 2 mm^3^ specimens, fixed using 2.5% glutaraldehyde, embedded with epoxy resin, and sectioned into ultrathin sections. Ultrastructural changes were observed using a transmission electron microscope (Ht7800, Hitachi).

### 2.13. Western Blotting

Total proteins were extracted from liver tissue samples, and the protein concentration was determined using the bicinchoninic acid (BCA) method. The standard western blotting method [27] was used with primary antibodies recognizing glutathione peroxidase 4 (GPX4; 1 : 500, ProteinTech), high mobility group box 1 (HMGB1; 1 : 500, ProteinTech), Toll-like receptor 4 (TLR4; 1 : 500, Santa Cruz Biotechnology), RELA proto-oncogene, NF-*κ*B Subunit (p65; 1 : 500, Cell Signaling Technology, Danvers, MA, USA), phosphorylated (p)-p65 (1 : 500, Cell Signaling Technology), IREB2 (1 : 200, Santa Cruz Biotechnology), FTH1 (1 : 500, Abcam), and TFR1 (1 : 200, Santa Cruz Biotechnology). Antibodies (1 : 800, Santa Cruz Biotechnology) detected *β*-actin as an internal reference protein. Chemiluminescence solution and an imaging system were used to detect the immunoreactive protein bands on the membrane. Image J 7.0 software (NIH, Bethesda, MD, USA) was used for semiquantitative analysis.

### 2.14. RNA Extraction and qRT-PCR

Total RNA was extracted from liver tissues, cells, and exosomes using the Trizol reagent (Takara, Shiga, Japan). The reverse transcription and quantitative real-time PCR (qPCR) steps of the qRT-PCR protocol were performed according to the manufacturer's instructions of the commercial kit (Takara, Shiga, Japan). *Actb* (encoding *β*-actin) was used as the internal normalization control for mRNA, and U6 was used as the normalization control for miRNAs. Detailed primer information is shown in Table S3 and Table S4.

### 2.15. Enzyme-Linked Immunosorbent Assay (ELISA)

Interleukin- (IL-) 6, tumor necrosis factor-alpha (TNF-*α*), and IL-1*β* levels in serum after steatotic liver IRI were detected according to the instructions of an ELISA kit (Multisciences Biotechnology Co., Ltd., Hangzhou, China).

### 2.16. Cell Immunofluorescence

SHPs after different treatments were fixed, Triton permeabilized, blocked using goat serum, and then incubated with primary antibodies recognizing IREB2 (1 : 200, Santa Cruz Biotechnology), FTH1 (1 : 200, Abcam), and TFR1 (1 : 200, Santa Cruz Biotechnology) at 4°C overnight, followed by incubation with secondary antibodies, and staining with 4′, 6-diamidino-2-phenylindole (DAPI). The cells were then observed under a fluorescence microscope.

### 2.17. Cell Viability

Cells were seeded in a 96-well plate, and then, a Cell Counting Kit-8 (CCK-8) (Solarbio) was used to detect cell viability after different pretreatment (5000 cells/well). The specific operation was carried out according to the manufacturer's instructions.

### 2.18. Lipid ROS Detection

C11-BODIPY (581/591), a lipid peroxidation sensor (5 *μ*M) (D3861, Invitrogen), was used to detect cellular lipid ROS. Pretreated SHPs were incubated in medium containing the lipid peroxidation sensor for 30 min, washed three times with PBS, and then, the BODIPY fluorescence intensity was detected using flow cytometry (Accuri C6 plus, Becton Dickinson, Franklin Lakes, NJ, USA).

### 2.19. Cell Death

Cell death was detected using propidium iodide (PI) staining (Keygen Biotech, Jiangsu, China). Cells were inoculated into 12 well plates at a density of 70–80%. After treatment with SHPs, cells (including floating dead cells) were collected and stained using 5 *μ*g/mL PI in the dark for 5 min. The percentage of PI positive dead cells was analyzed using flow cytometry (Accuri C6 plus) and an FL2 (peridinin-chlorophyll-protein complex (PerCP)) detector.

### 2.20. Mitochondrial Function

The cell mitochondrial membrane potential was detected using a JC-1 mitochondrial membrane potential detection kit (Beyotime). The pretreated cells were incubated with JC-1 working solution at 37°C for 20 min, washed twice with JC-1 buffer, detected under a fluorescence microscope (IX71, Olympus), and analyzed using flow cytometry (Accuri C6 plus).

### 2.21. Statistical Analysis

Graph pad Prism 8.0 (GraphPad Software Inc., San Diego, CA, USA) was used for statistical analysis. The measured data conformed to the normal distribution and are expressed as the mean ± standard deviation. An unpaired Student's *t*-test was used for comparisons between two groups, and one-way analysis of variance (ANOVA) was used to compare data among three groups. *P* < 0.05 was regarded as statistically significant.

## 3. Results

### 3.1. Identification of the Morphological and Biological Characteristics of HO-1/BMMSCs

Under light microscope, HO-1/BMMSCs showed the typical spindle appearance of BMMSCs (Figure S1A) and showed osteogenic and adipogenic multidirectional differentiation potential (Figures S1B, S1C). Flow cytometry showed that almost 99% of the HO-1/BMMSCs were positive for CD29, CD90, and RT1A, and similarly, approximately 99% of the HO-1/BMMSCs were negative for CD34, CD45, and RT1B (Figures S1D-S1F). These results showed that transfection of HO-1 gene and the consequent increase in HO-1 levels (Figures S1G-S1I) did not change the basic biological characteristics of the BMMSCs.

### 3.2. The Steatotic Liver IRI Model and HO-1/BMMSC Colonization in Liver

After continuous feeding of rats with a high-fat diet for 20 weeks, HE staining (Figure S2A) showed a mixed macrovesicular region in the liver, and the steatotic area was more than 60%. Meanwhile, oil red O staining (Figure S2B) showed obvious lipid deposition and diffuse distribution of red particles, indicating the successful establishment of a severely steatotic liver.

In the IRI model (Figure S2C), before ischemia, the normal liver was ruddy, with sharp edges, and the steatotic liver was light yellow with blunt edges. Immediately after ischemia, the left lobe and middle lobe of the liver became pale. During reperfusion, the recovery of liver blood flow showed patchy changes, indicating that the IRI model was established successfully.

At 24 h after reperfusion, animal *in vivo* imaging (Figure S2D) showed that GFP/HO-1/BMMSCs had colonized in the liver, and analysis of frozen sections showed that the number of HO-1/BMMSCs colonizing the liver (Figure S2E) was more than that of BMMSCs, suggesting that HO-1/BMMSCs had a prolonged survival time in the liver compared with BMMSCs.

### 3.3. Ferroptosis Is Involved in Steatotic Liver IRI

**Figure 1:**
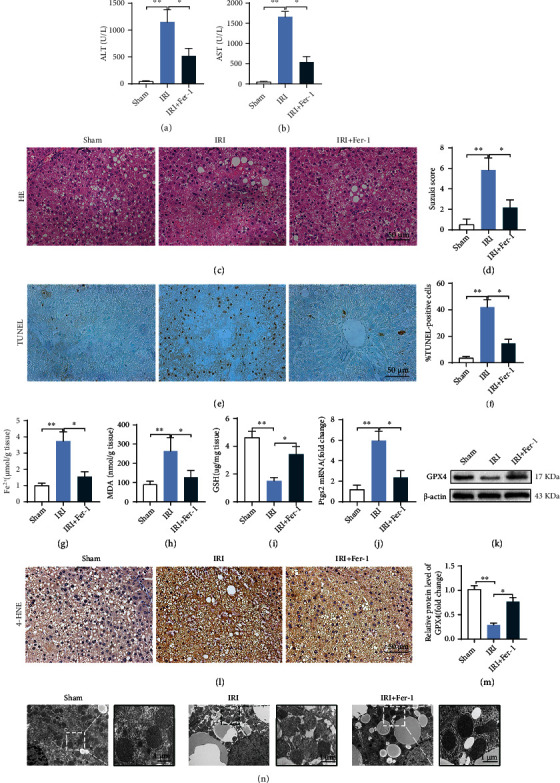
Inhibition of ferroptosis can reduce steatotic liver IRI. Serum ALT (a) and AST (b) levels in IRI, with or without Fer-1 treatment. (c) Pathological HE staining of steatotic liver IRI; Fer-1 significantly reduced liver pathological injury. (d) Suzuki score of steatotic liver IRI. (e) TUNEL staining of steatotic liver IRI; Fer-1 significantly decreased the number of TUNEL positive cells (f). The contents of Fe^2+^ (g), MDA (h), and GSH (i) in IRI, with or without Fer-1, were detected using colorimetry. (j) The *Ptgs2* mRNA level. (k) GPX4 protein level determined using western blotting. (l) Representative images of 4-HNE immunohistochemical staining. (m) Semiquantitative analysis of (k). (n) Liver transmission electron microscopy showing that the number of mitochondrial cristae decreased, the membrane density increased, and membrane was ruptured in steatotic liver IRI and was improved by Fer-1. *n* = 6 per group. Data are presented as the mean ± SEM. ^∗^*P* < 0.05; ^∗∗^*P* < 0.01.

The liver function evaluation index showed that compared with the Sham group, the levels of serum ALT (Figure 1(a)) and AST (Figure 1(b)) in the IRI group were significantly higher, HE staining (Figures 1(c) and 1(d)) showed that the liver had marked histopathological damage, which was characterized by obvious congestion and expansion of hepatic sinuses, with a large area of homogeneous red staining, unstructured coagulation necrosis, and a higher Suzuki score. Concurrently, the percentage of TUNEL positive cells increased significantly (Figures 1(e) and 1(f)). However, after Fer-1 treatment, the above liver function indexes improved significantly compared with those in the IRI group.

Ferroptosis evaluation showed that the Fe^2+^ level (Figure 1(g)) and MDA content (Figure 1(h)) increased and the GSH content (Figure 1(i)) decreased in the IRI group. At the same time, *Ptgs2* mRNA (encoding prostaglandin-endoperoxide synthase 2) expression (Figure 1(j)) and the 4-HNE level (Figure 1(l)) were significantly higher than those in the Sham group, while the GPX4 level (Figures 1(k) and 1(m)) was significantly lower than in the Sham group. In addition, TEM (Figure 1(n)) showed that the number of mitochondrial cristae decreased, the membrane density increased, and membrane rupture occurred in the IRI group. After treatment with Fer-1, the above ferroptosis indexes improved significantly. These results indicated that ferroptosis is involved in steatotic liver IRI, and inhibiting ferroptosis could reduce the extent of the injury. In addition, we found that Fe^2+^ (Figure S3A) and MDA levels (Figure S3B) were significantly higher, but the GPX4 protein level (Figure S3C) was lower in steatotic liver IRI compared with those in normal liver IRI. Moreover, ALT (Figure S3D) and AST (Figure S3E) levels were higher in the steatotic liver, suggesting that ferroptosis might be an important mechanism of serious liver tissue injury after steatotic liver IRI.

### 3.4. HO-1/BMMSCs Can Protect against Steatotic Liver IRI

**Figure 2:**
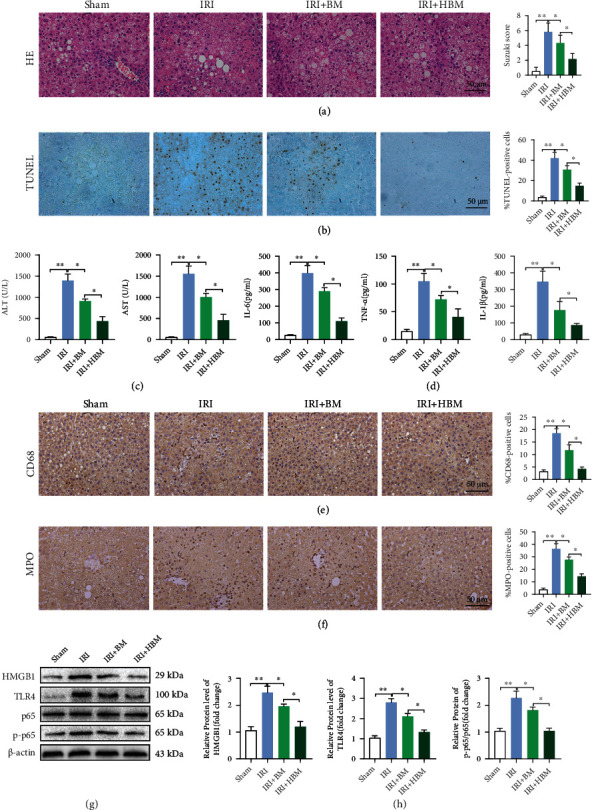
HO-1/BMMSCs attenuate histopathological injury and hepatocyte death and suppress the activation of the innate immune response in steatotic liver IRI. (a) Pathological HE staining of steatotic liver IRI with BM or HBM treatment. (b) TUNEL staining of steatotic liver IRI with BM or HBM treatment. (c) Serum ALT and AST levels in IRI with BM or HBM treatment. (d) IL-6, TNF-*α*, and IL-1*β* levels detected using ELISA. Representative images of CD68 (e) and MPO (f) immunohistochemical staining. (g) HMGB1/TLR4/p-p65 protein levels assessed using western blotting. (h) Semiquantitative analysis of (g). BM, BMMSCs; HBM, HO-1/BMMSCs. *n* = 6 per group. Data are presented as the mean ± SEM. ^∗^*P* < 0.05; ^∗∗^*P* < 0.01.

HE (Figure 2(a)) and TUNEL staining (Figure 2(b)) showed that the addition of HO-1/BMMSCs significantly reduced the liver histopathological injury and hepatocyte death compared with BMMSC treatment alone and reduced the Suzuki score and the percentage of TUNEL positive staining. In addition, HO-1/BMMSCs reduced the levels of ALT, AST (Figure 2(c)), and proinflammatory cytokines significantly (Figure 2(d)), including IL-6, TNF-*α*, and IL-1*β*.

In addition, we evaluated the activation of the innate immune response in steatotic liver IRI. Immunohistochemistry showed that CD68 (a macrophage marker) and MPO (a neutrophil marker) levels increased significantly in the IRI group, while the addition of HO-1/BMMSCs decreased the percentage of CD68 (Figure 2(e)) and MPO (Figure 2(f)) positive cells significantly compared with BMMSC treatment alone. Moreover, the levels of HMGB1, TLR4, and p-p65 increased significantly in the IRI group. After HO-1/BMMSCs intervention, the HMGB1, TLR4, and p-p65 levels (Figures 2(g) and 2(h)) decreased significantly, and the effect was better than that of BMMSC treatment alone.

The results showed that HO-1/BMMSCs could protect steatotic livers from IRI significantly.

### 3.5. HO-1/BMMSCs Can Inhibit Ferroptosis in Steatotic Liver IRI

To explore whether the protective effect of HO-1/BMMSCs on steatotic liver IRI is related to the regulation of ferroptosis, we evaluated the effect of HO-1/BMMSCs on ferroptosis indicators.

**Figure 3:**
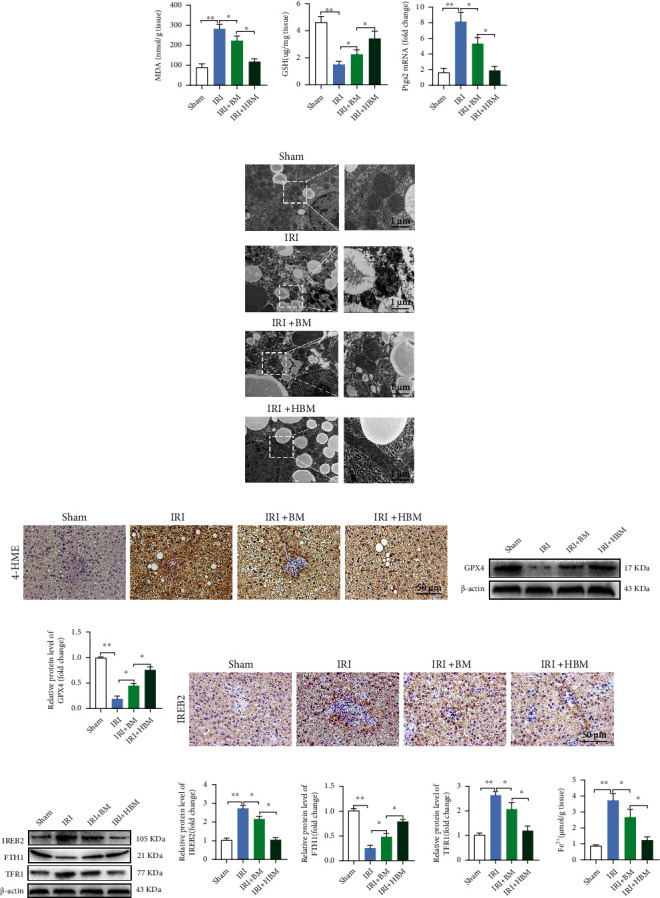
HO-1/BMMSCs could suppress ferroptosis of steatotic liver IRI and this modulation could be mediated by IREB2. Evaluation of ferroptosis in steatotic liver IRI with BMMSCs or HO-1/BMMSC treatment. (a) MDA content detected using colorimetry. (b) GSH content detected using colorimetry. (c) The *Ptgs2* mRNA level. (d) Representative TEM images showing that HO-1/BMMSCs improved the ferroptosis morphology of mitochondria (mitochondrial cristae decrease, membrane density increased, and membrane rupture). (e) Representative immunohistochemical images of 4-HNE. (f) GPX4 protein levels assessed using western blotting. (g) Semiquantitative analysis of (f). (h) Representative immunohistochemical images of IREB2 showing that that positive expression was increased significantly in IRI and decreased by HO-1/BMMSC treatment. (i) IREB2/FTH1/TFR1 protein levels assessed using western blotting. (j) Semiquantitative analysis of (i). (k) The Fe^2+^ level detected using colorimetry. *n* = 6 per group. Data are presented as the mean ± SEM. ^∗^*P* < 0.05; ^∗∗^*P* < 0.01.

Ferroptosis indexes showed that compared with the Sham group, ferroptosis occurred significantly in IRI, and after HO-1/BMMSC treatment, the content of MDA (Figure 3(a)) decreased, GSH increased (Figure 3(b)), and the *Ptgs2* mRNA level (Figure 3(c)) decreased. In addition, the ferroptosis-related morphology of the mitochondria (Figure 3(d)) improved, the level of 4-HNE was downregulated (Figure 3(e)), and the level of GPX4 (Figures 3(f) and 3(g)) was upregulated. The results showed that HO-1/BMMSCs could inhibit ferroptosis in steatotic liver IRI. Therefore, HO-1/BMMSCs could protect against steatotic liver IRI injury by regulating ferroptosis.

### 3.6. HO-1/BMMSC-Induced Ferroptosis Modulation Could Be Mediated by IREB2

IREB2 is the main regulator of cellular iron homeostasis. Studies have shown that IREB2 is related to the increase in Fe^2+^ in the steatotic liver and liver IRI [15, 17]. Moreover, *IREB2* is a marker gene for ferroptosis. When ferroptosis occurs, the level of *IREB2* mRNA increases and aggravates ferroptosis. Knockdown of *IREB2* can reduce ferroptosis [11, 20]. Therefore, ferroptosis in steatotic liver IRI is likely to be related to the abnormal expression of *IREB2*. Therefore, we also examined the regulatory effect of HO-1/BMMSCs on *Ireb2* expression.

Compared with that in the Sham group, the immunohistochemical positive rate (Figure 3(h)) and protein level (Figures 3(i) and 3(j)) of IREB2 in IRI were increased significantly, resulting in a decrease in FTH1 levels, an increase in TFR1 levels, and finally, an increased Fe^2+^ level (Figure 3(k)). However, after treatment with HO-1/BMMSCs, the level of IREB2 decreased significantly, the level of FTH1 increased, the level of TFR1 decreased, the Fe^2+^ content decreased, and these effects were significantly better than those of BMMSCs alone. These results suggested that the increased level of IREB2 in steatotic liver IRI might be the cause of the increase in ferroptosis. In addition, regulating the expression of IREB2 might be one of the mechanisms by which HO-1/BMMSCs inhibit ferroptosis in steatotic liver IRI.

### 3.7. Ferroptosis Is Involved in the SHP-HR Model in Which IREB2 Expression Is Increased

To confirm the preliminary results obtained in the rat steatotic liver IRI model and further explore the specific mechanism, we induced steatosis of IAR20 cells and established a serum-free HR model of SHP *in vitro* to simulate ischemia-reperfusion *in vivo*.

Ferroptosis-related indexes showed that compared with the Sham group, in the HR group, the Fe^2+^ level (Figure S4A) and MDA content (Figure S4B) increased, the GSH content (Figure S4C) decreased, the *Ptgs2* mRNA (Figure S4D) and Lipid ROS (Figures S4E, S4F) levels increased, while the level of GPX4 (Figures S4G, S4H) decreased. However, the above ferroptosis indexes improved significantly after Fer-1 intervention. Meanwhile, cell viability (Figure S4I), mitochondrial membrane potential dysfunction (Figure S4J), and cell death in the HR group (Figures S4K, S4L) were also significantly improved by Fer-1. At the same time, we compared the protective effect of Fer-1 and an apoptosis inhibitor (Emricasan) on SHP-HR cell viability (Figure S4I). The results showed that Emricasan had limited effect on improving SHP-HR cell viability, and the protective effect of Fer-1 was significantly better than that of Emricasan, indicating that ferroptosis plays a more important role in SHP-HR. The above results showed that ferroptosis is involved in SHP-HR. Inhibiting ferroptosis could reduce the cell damage, which is consistent with the results of the *in vivo* IRI model, and better simulates the actual situation *in vivo*.

In addition, we found that in the SHP-HR group, the mRNA and protein levels of IREB2 increased, the mRNA and protein levels of FTH1 decreased, and those of TFR1 increased (Figures S4M-S4O).

Both the *in vivo* and *in vitro* results confirmed that IREB2 expression was elevated, which indicated IREB2 might play an important role in ferroptosis and tissue-cell injury in steatotic liver IRI and SHP-HR.

### 3.8. Knockdown of Ireb2 in SHPs Reduces the Level of Fe^2+^, Inhibits Ferroptosis, and Alleviates Cell Injury in SHP-HR

To verify the role of IREB2 in ferroptosis and cell injury of SHP-HR, we used si-RNA technology to knock down the *Ireb2* mRNA level in SHPs and then established the HR model. Compared with that in the Sham and si-NC groups, si-IREB2 reduced the level of IREB2 mRNA and protein in SHPs significantly, increased the mRNA and protein expression of FTH1, and decreased the expression of TFR1 (Figures S5A-S5C), indicating that *Ireb2* knockdown in SHPs was successful.

**Figure 4:**
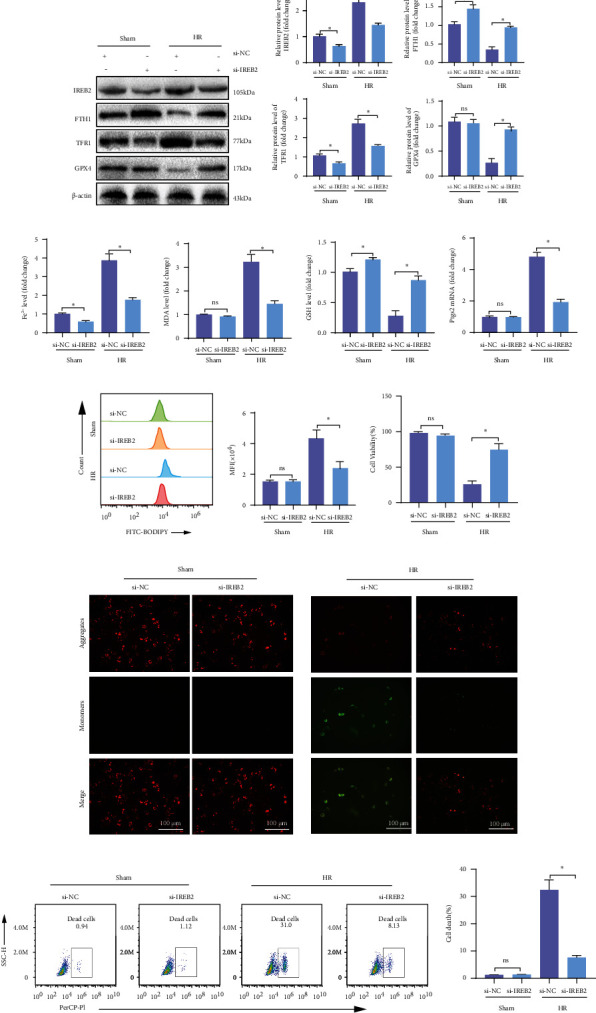
Knockdown of *Ireb2* in SHPs can reduce the level of Fe^2+^, inhibit ferroptosis, and alleviate cell injury in SHP-HR. Evaluation of ferroptosis and cell injury in SHP-HR with si-NC or si-IREB2. (a) IREB2/FTH1/TFR1 protein levels assessed using western blotting. (b) Semiquantitative analysis of (a). (c) The Fe^2+^ level detected using colorimetry. (d) The MDA content detected using colorimetry. (e) The GSH content detected using colorimetry. (f) The *Ptgs2* mRNA level. (g) The lipid ROS level detected using C11-BODIPY and flow cytometry. (h) Quantitative analysis of C11-BODIPY fluorescence intensity. (i) Cell viability detected using a CCK-8 kit. (j) Cell mitochondrial membrane potential detected using JC-1 staining and fluorescence microscopy, showing that the JC-1 monomer (green) increased and the polymer (red) decreased in HR with si-NC, representing mitochondrial membrane potential dysfunction, while si-IREB2 reversed this change and improved the mitochondrial membrane potential. (k) Cell death ratio detected using PI staining and flow cytometry. (l) Quantitative analysis of (k). *n* = 3 per group. Data are presented as the mean ± SEM. ^∗^*P* < 0.05; ^∗∗^*P* < 0.01.

In the HR model, compared with that in the si-NC group, si-IREB2 significantly decreased the IREB2 protein level, increased the FTH1 and GPX4 protein levels, decreased the TFR1 protein level (Figures 4(a) and 4(b)), and decreased the Fe^2+^ level (Figure 4(c)). In addition, after si-IREB2 treatment, the MDA content (Figure 4(d)) decreased, the GSH content (Figure 4(e)) increased, and the *Ptgs2* mRNA (Figure 4(f)) and Lipid ROS (Figures 4(g) and 4(h)) levels were downregulated. In terms of cell injury, si-IREB2 significantly improved cell viability (Figure 4(i)) and mitochondrial membrane potential dysfunction (Figure 4(j)) and decreased the proportion of cell death (Figures 4(k) and 4(l)). In conclusion, IREB2 plays an important role in ferroptosis and cell injury in SHP-HR.

### 3.9. HO-1/BMMSCs Could Regulate the IREB2 Protein Level, Inhibit Ferroptosis, and Alleviate Cell Injury In SHP-HR

**Figure 5:**
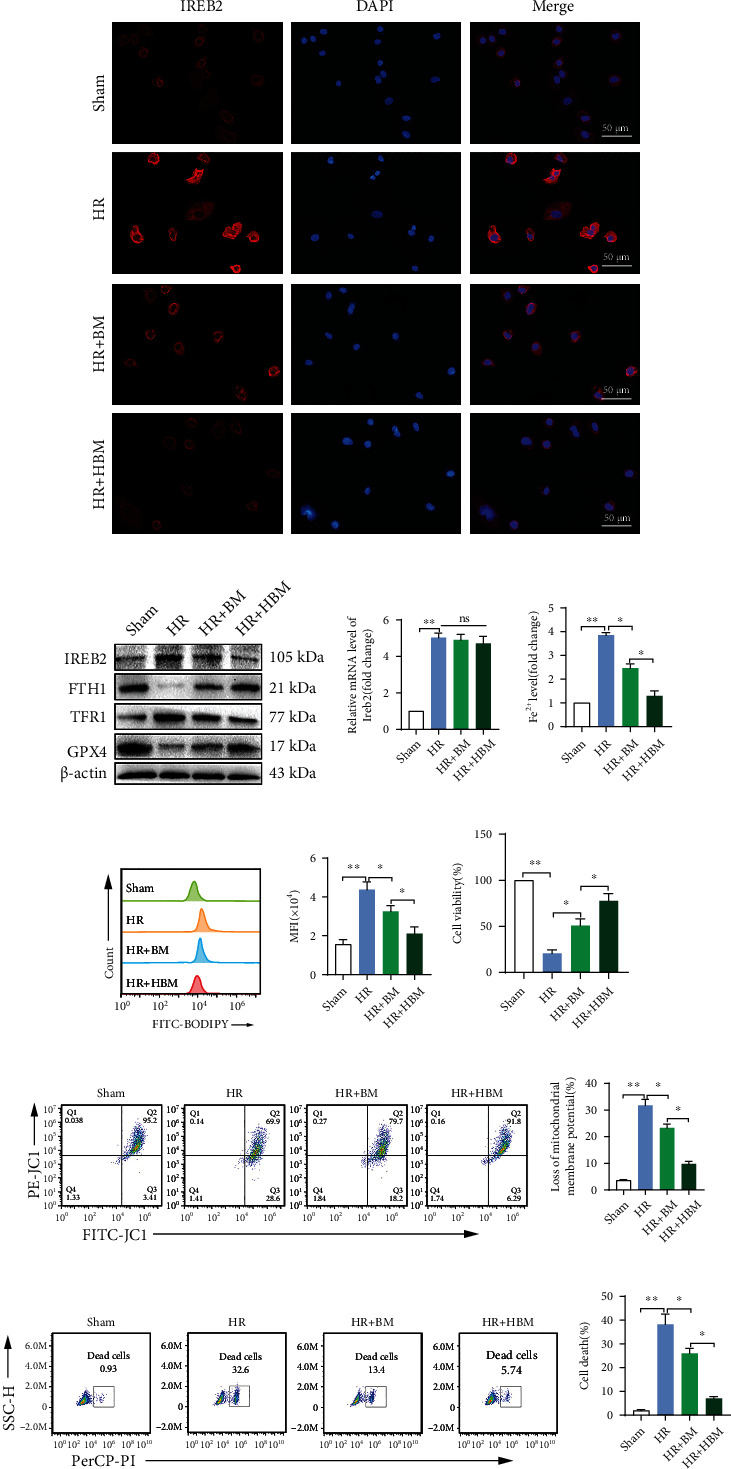
HO-1/BMMSC-mediated inhibition of ferroptosis and alleviation of SHP-HR injury could be mediated by regulating the protein levels of IREB2. Evaluation of ferroptosis and cell injury in SHP-HR with BMMSCs or HO-1/BMMSCs. (a) Representative immunofluorescence images of IREB2 showing that positive expression increased significantly in HR and was decreased by HO-1/BMMSC treatment. (b) IREB2/FTH1/TFR1/GPX4 protein levels assessed using western blotting. (c) *Ireb2* mRNA level detected using qRT-PCR. (d) Fe^2+^ level detected using colorimetry. (e) The lipid ROS level detected using C11-BODIPY and flow cytometry. (f) Quantitative analysis of C11-BODIPY fluorescence intensity. (g) Cell viability detected using a CCK-8 kit. (h) Cell mitochondrial membrane potential detected using JC-1 staining and flow cytometry. (i) Quantitative analysis of (h). (j) Cell death ratio detected using PI staining and flow cytometry. (k) Quantitative analysis of (j). *n* = 3 per group. Data are presented as the mean ± SEM. ^∗^*P* < 0.05; ^∗∗^*P* < 0.01.

In view of the important role of IREB2 in ferroptosis and cell injury in SHP-HR, and based on the above verification, we further explored whether HO-1/BMMSCs could inhibit ferroptosis and protect cell injury by regulating IREB2 in SHP-HR. Consistent with the results *in vivo*, HO-1/BMMSC treatment significantly reduced IREB2 protein levels in the SHP-HR group, and the effect was significantly better than that of BMMSCs (Figures 5(a) and 5(b)), but had no significant effect on the mRNA level of *Ireb2* (Figures 5(c)). At the same time, HO-1/BMMSCs increased the protein level of FTH1 and reduced the protein level of TFR1 significantly, which are both posttranscriptionally regulated by IREB2 (Figure 5(b)), and finally reduced the Fe^2+^ level (Figure 5(d)). These results indicated that HO-1/BMMSCs could reduce the level of Fe^2+^ by regulating IREB2. Furthermore, we found that HO-1/BMMSCs significantly reduced the lipid ROS level (Figures 5(e) and 5(f)), indicating that HO-1/BMMSCs inhibited the ferroptosis of the SHP-HR group. Meanwhile, HO-1/BMMSCs significantly improved cell viability (Figure 5(g)), mitochondrial membrane potential dysfunction (Figures 5(h) and 5(i)), and the cell death ratio in the SHP-HR group (Figures 5(j) and 5(k)).

These results showed that HO-1/BMMSCs could inhibit ferroptosis and alleviate cell injury in SHP-HR, and this effect could be mediated by regulating the protein levels of IREB2.

### 3.10. Exosomes Derived from HO-1/BMMSCs Dominate the Role of HO-1/BMMSCs in Regulating IREB2, Inhibiting Ferroptosis, and Alleviating Cell Injury in SHP-HR

Studies have shown that exosomes are important paracrine mediators of BMMSCs' function and can reduce liver IRI [8, 9]. Therefore, we explored whether the role of HO-1/BMMSCs in regulating IREB2, inhibiting ferroptosis, and protecting against SHP-HR injury is mainly mediated by their exosomes.

First, to evaluate whether exosomes are the main mechanism by which HO-1/BMMSCs exert their function in the SHP-HR model, we treated HO-1/BMMSCs with the exosome inhibitor, GW4869 (GW). The results showed that compared with HO-1/BMMSCs, HO-1/BMMSCs treated with GW significantly weakened the regulation effect of IREB2 protein level (Figure S6A), resulting in an increase in Fe^2+^ (Figure S6B) and lipid ROS levels (Figure S6C), and a decrease in cell viability (Figure S6D), which significantly weakened the effect of HO-1/BMMSCs on inhibiting ferroptosis and alleviating cell injury in SHP-HR. Thus, exosomes seem to be the main mechanism of HO-1/BMMSCs' function in SHP-HR model.

**Figure 6:**
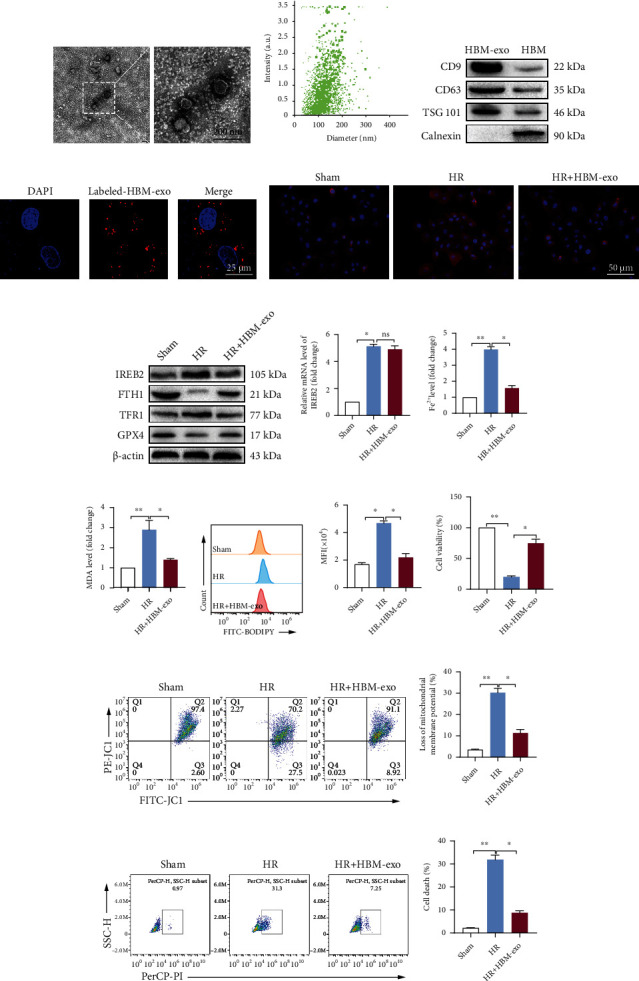
HO-1/BMMSC-derived exosomes inhibit ferroptosis and protect SHP-HR injury by regulating IREB2. (a) HBM-exos were observed as vesicles using transmission electron microscope. (b) Nanoparticle tracking analysis showing that the particle size of the HBM-exos was concentrated in the 100–150 nm range. (c) HBM-exos were positive for CD63, TSG101, and CD9 proteins, but negative for calnexin. (d) Confocal fluorescence microscopy showing that SHPs ingested CM-DiI- (red-) labeled HBM-exos. Evaluation of ferroptosis and cell injury in SHP-HR, with or without HBM-exos. (e) Representative immunofluorescence images of IREB2 showing that HBM-exos downregulated the protein level of IREB2. (f) IREB2/FTH1/TFR1/GPX4 protein levels assessed using western blotting. (g) *Ireb2* mRNA level detected using qRT-PCR. (h) The Fe^2+^ levels detected using colorimetry. (i) The MDA content detected using colorimetry. (j) The lipid ROS level detected using C11-BODIPY and flow cytometry. (k) Quantitative analysis of C11-BODIPY fluorescence intensity. (l) Cell viability detected using a CCK-8 kit. (m) Cell mitochondrial membrane potential detected using JC-1 staining and flow cytometry. (n) Quantitative analysis of (m). (o) Cell death ratio detected using PI staining and flow cytometry. (p) Quantitative analysis of (o). HBM-exos, HO-1/BMMSC exosomes. *n* = 3 per group. Data are presented as the mean ± SEM. ^∗^*P* < 0.05; ^∗∗^*P* < 0.01.

Next, we extracted exosomes from HO-1/BMMSCs to further study the protective effects and molecular mechanism of exosomes in regulating the IREB2 protein level, inhibiting ferroptosis, and alleviating cell injury in SHP-HR. HO-1/BMMSC exosomes (HBM-exos) were isolated from HO-1/BMMSCs by ultracentrifugation. Exosomes were verified by transmission electron microscopy (Figure 6(a)), NTA particle size analysis (Figure 6(b)), and exosome specific proteins (CD9, CD63, TSG101, and calnexin, in which calnexin was used as the negative control) (Figure 6(c)). CM-DiI labeled HBM-exos were cocultured with SHPs for 6 h, and then, the uptake of HBM-exos by SHPs was observed using confocal fluorescence microscopy (Figure 6(d)).

The results were similar to those of HO-1/BMMSCs: HBM-exos significantly decreased the protein level of IREB2 in the SHP-HR group (Figures 6(e) and 6(f)), but had no significant effect on the mRNA level of *Ireb2* (Figure 6(g)). Meanwhile, HBM-exos upregulated the protein levels of FTH1 and GPX4, downregulated the level of TFR1 (Figure 6(f)), and decreased the Fe^2+^ level (Figure 6(h)). Finally, HBM-exos decreased the content of MDA (Figure 6(i)) and downregulated the level of lipid ROS (Figures 6(j) and 6(k)). In addition, the cell injury index showed that HBM-exos significantly improved cell viability (Figure 6(l)), reduced the dysfunction of mitochondrial membrane potential (Figures 6(m) and 6(n)), and reduced the proportion of cell death (Figures 6(o) and 6(p)). In conclusion, HBM-exos could reduce the level of Fe^2+^ and ferroptosis by regulating IREB2, thus protect against SHP-HR injury.

Therefore, HBM-exos might represent an important mechanism by which HO-1/BMMSCs inhibit ferroptosis and protect against SHP-HR injury.

### 3.11. miR-29a-3p, Which Targeted IREB2, Is Abundant in HBM-exos and Could Decrease IREB2 Protein Expression and Inhibit Ferroptosis

To further identify the exosomal components responsible for HBM-exos-regulation of IREB2 and the inhibition of ferroptosis, we analyzed exosomal miRNAs targeting IREB2.

**Figure 7:**
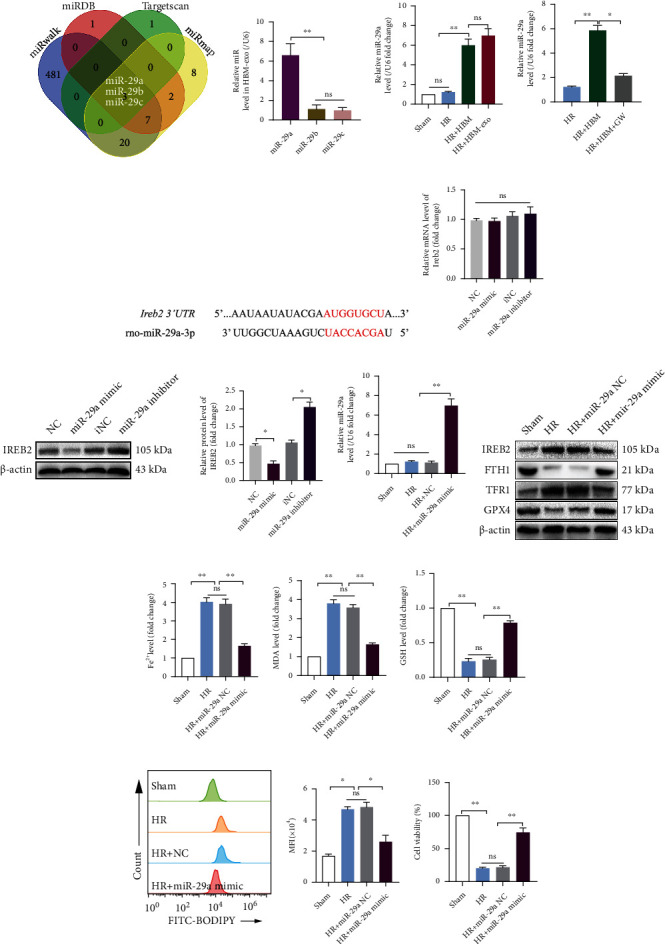
miR-29a-3p, which targets *Ireb2*, is abundant in HBM-exos and could decrease IREB2 protein levels and inhibit ferroptosis. (a) miRNAs targeting the 3′-UTR of *Ireb2* mRNA were predicted by TargetScan, miRWalk, miRDB, and miRmap databases. Finally, miR-29a-3p, miR-29b-3p, and miR-29c-3p were screened out using the four databases. (b) The level of miR-29a-3p was highest in HBM-exos. (c) The level of miR-29a-3p increased significantly in HBM-exos or HBM with the SHP-HR coculture system. (d) The miR-29a-3p level in the SHP-HR coculture system decreased significantly when HO-1/BMMSCs were treated with GW. (e) Pairing sequence of miR-29a-3p and 3′-UTR of *Ireb2*. (f) Neither the mimic nor the inhibitor of miR-29a-3p had a significant effect on the *Ireb2* mRNA level. (g) The miR-29a-3p mimic significantly decreased the protein level of IREB2, and the miR-29a-3p inhibitor significantly increased the protein level of IREB2. (h) The level of miR-29a-3p in the HR + miR − 29a − 3p mimic group was significantly higher than that in the Sham, HR, and HR + NC groups. (i) IREB2/FTH1/TFR1/GPX4 protein levels detected using western blotting. (j) The Fe^2+^ level detected using colorimetry. (k) MDA content detected using colorimetry. (l) GSH content detected using colorimetry. (m) The lipid ROS level detected using C11-BODIPY and flow cytometry. Quantitative analysis of C11-BODIPY fluorescence intensity is shown on the right. (n) Cell viability detected using a CCK-8 kit. *n* = 3 per group. Data are presented as the mean ± SEM. ^∗^*P* < 0.05; ^∗∗^*P* < 0.01.

The miRNAs targeting the 3′-UTR of rat *Ireb2* mRNA were predicted using TargetScan, miRWalk, miRDB, and miRmap databases. Finally, miR-29a-3p, miR-29b-3p, and miR-29c-3p were screened from the four databases (Figure 7(a)). Meanwhile, the level of miR-29a-3p was the highest in HBM-exos (Figure 7(b)), and miR-29a-3p levels increased significantly in HBM-exos or HBMs cocultured with SHP-HR cells, while there was no significant change in the Sham and HR groups (Figure 7(c)). The results showed that HBM-exos were rich in miR-29a-3p. Meanwhile, we found that the miR-29a-3p level in the SHP-HR coculture system decreased significantly when HO-1/BMMSCs were treated with GW (Figure 7(d)), which indicated that miR-29a-3p mainly comes from exosomes of HO-1/BMMSCs. At the same time, database prediction showed that miR-29a-3p is conservatively paired with the 3′-UTR region of the *Ireb2* mRNA (Figure 7(e)).

To verify that miR-29a-3p can target *Ireb2* and affect its expression level, miR-29a-3p mimic and inhibitor were used to detect its effect on IREB2 mRNA and protein expression in SHPs. The results showed that the miR-29a-3p mimic and inhibitor did not significantly affect the level of *Ireb2* mRNA (Figure 7(f)); however, the miR-29a-3p mimic significantly decreased the IREB2 protein level, while the miR-29a-3p inhibitor significantly increased the IREB2 protein level (Figure 7(g)), indicating that miR-29a-3p could reduce the protein level of IREB2 by posttranscriptional regulation mechanism.

The effect of the miR-29a-3p mimic on SHP-HR was further explored. The results showed that the level of miR-29a-3p in the HR + miR − 29a − 3p mimic group was significantly higher than that in Sham, HR, and HR + NC group (Figure 7(h)). At the same time, the IREB2 protein level decreased significantly, the FTH1 and GPX4 protein levels increased, the TFR1 protein level decreased (Figure 7(i)), and the Fe^2+^ level (Figure 7(j)) decreased in the SHP-HR group. In addition, in the HR + miR − 29a − 3p mimic group, there was a significantly decreased MDA content (Figure 7(k)), an increased GSH content (Figure 7(l)), a decreased lipid ROS level (Figure 7(m)), and improved cell viability (Figure 7(n)).

### 3.12. Loss of miR-29a-3p in HBM-exos Results in Loss of Regulation of IREB2-Mediated Ferroptosis and the Protective Function for SHP-HR

**Figure 8:**
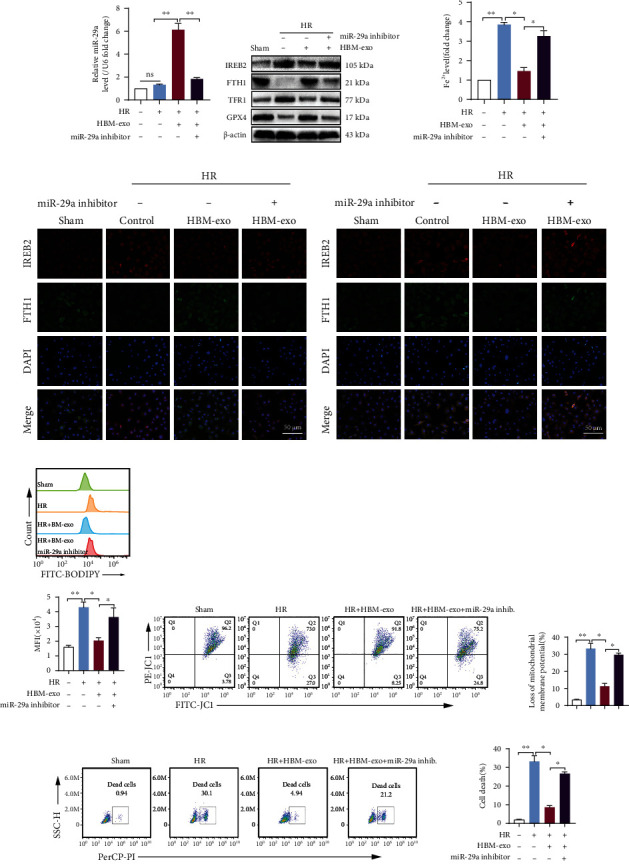
Loss of miR-29a-3p in HBM-exos results in loss of regulation of IREB2-mediated ferroptosis and the protective function of SHP-HR. Evaluation of ferroptosis and cell injury in HBM − exos + SHP − HR, with or without the miR-29a-3p inhibitor treatment. (a) The miR-29a-3p inhibitor significantly reduced the elevated level of miR-29a-3p in the coculture system of HBM-exos and SHP-HR. (b) IREB2/FTH1/TFR1/GPX4 protein levels assessed using western blotting, showing that miR-29a-3p inhibitor eliminated the regulation by HBM-exos on the protein levels of IREB2/FTH1/TFR1/GPX4. (c) The Fe^2+^ level detected using colorimetry. (d) Representative images of IREB2 and FTH1 immunofluorescence costaining. (e) Representative images of IREB2 and TFR1 immunofluorescence costaining. (f) Lipid ROS levels detected using C11-BODIPY and flow cytometry. (g) Cell mitochondrial membrane potential detected using JC-1 staining and flow cytometry. (h) Quantitative analysis of (g). (i) Cell death ratio detected using PI staining and flow cytometry. (j) Quantitative analysis of (i). *n* = 3 per group. Data are presented as the mean ± SEM. ^∗^*P* < 0.05; ^∗∗^*P* < 0.01.

To prove that miR-29a-3p is an important component of HBM-exos in regulating ferroptosis and alleviating SHP-HR injury, we transfected the miR-29a-3p inhibitor into HBMs and then isolated exosomes, which were used to intervene in SHP-HR. The results showed that the HBM-exos upregulated the level of miR-29a-3p in the SHP-HR group, while the level of miR-29a-3p decreased after transfection of the miR-29a-3p inhibitor (Figure 8(a)). At the same time, after transfection of the miR-29a-3p inhibitor, the reduced IREB2 protein level in the coculture system of HBM-exos with SHP increased, the FTH1 protein level decreased, and the TFR1 protein level and Fe^2+^ level increased (Figures 8(b)–8(e)). In addition, the miR-29a-3p inhibitor weakened the inhibitory effect of HBM-exos on ferroptosis of the SHP-HR group, leading to an increase in the lipid ROS level (Figure 8(f)) and weakened the protective effect of HBM-exos on mitochondrial membrane potential dysfunction (Figures 8(g) and 8(h)) and cell death (Figures 8(i) and 8(j)).

The results showed that HBM-exos' regulation of IREB2 mainly depends on their secreted miR-29a-3p, so as to reduce the level of intracellular Fe^2+^, inhibit ferroptosis, and protect against SHP-HR injury.

## 4. Discussion

In the present study, we showed that HO-1/BMMSCs have a protective effect on rat steatotic liver IRI and SHP-HR. At least part of the protective mechanism comprises inhibiting ferroptosis. In addition, we demonstrated that the mechanism by which HO-1/BMMSCs inhibit ferroptosis involves the extracellular transfer of HO-1/BMMSC-derived exosomes to SHPs and relies on miR-29a-3p in the exosomes to interfere with IREB2-related intracellular iron homeostasis signaling.

Iron, lipid, and antioxidant metabolisms play an important role in the process of ferroptosis. Among them, the increase of intracellular Fe^2+^ level is the key factor to induce large accumulation of lipid peroxide and promote ferroptosis [12]. Studies have shown that the increase in Fe^2+^ is an important cause of the occurrence and development of a steatotic liver and liver IRI, and inhibiting ferroptosis can reduce the progression of nonalcoholic fatty liver and liver IRI [13, 14]. Our *in vivo* and *in vitro* studies found that inhibition of ferroptosis by Fer-1 can improve liver function, reduce liver pathological injury, increase cell viability, and reduce the proportion of cell injury death, suggesting that ferroptosis is involved in the steatotic liver IRI and SHP-HR.

Our previous study confirmed that HO-1/BMMSCs could protect rat normal liver against IRI by inhibiting the inflammatory response [7]. In this study, we found that HO-1/BMMSCs could also protect against steatotic liver IRI and SHP-HR. Compared with BMMSCs, HO-1/BMMSCs improved the histopathological damage of the steatotic liver and reduced the proportion of cell death to a greater extent. At the same time, HO-1/BMMSCs inhibited the activation of the innate immune response to IRI, such as the activation of the HMGB1/TLR4/p-p65 inflammatory pathway and the activation and recruitment of macrophages and neutrophils. In addition, HO-1/BMMSCs downregulated the Fe^2+^ level and the lipid peroxidation index of ferroptosis *in vivo* and *in vitro*, upregulated the GSH content and GPX4 protein level, and improved the mitochondrial signs of ferroptosis. These results suggested that the regulation of ferroptosis might be one of the important mechanisms by which HO-1/BMMSCs play a protective role.

Nonalcoholic fatty liver is often accompanied by an increase in the liver iron load and an increase in Fe^2+^ level during liver IRI has been reported [15, 17]. Therefore, the cause of ferroptosis in the steatotic liver and liver IRI is related to an increased Fe^2+^ level. However, intracellular iron homeostasis is mainly mediated by IREB2, which regulates the posttranscriptional processing of iron metabolism-related proteins [18, 19]. IREB2 binding to the iron response element (IRE) in the 5′-UTR region of *FTH1* mRNA inhibited its protein translation, while binding to the IRE in the 3′-UTR region of *TFR1* mRNA stabilized its mRNA transcript and prevented its rapid degradation, resulting in an increase in intracellular Fe^2+^ [28].

As the main regulator of intracellular iron supply, IREB2 affects cell energy production and the redox state, and its overexpression leads to mitochondrial damage and general oxidative stress [29, 30]. More importantly, *IREB2* has been proven to be a marker gene of ferroptosis. When ferroptosis occurs, its mRNA expression increases and promotes the occurrence of ferroptosis. Knocking down *IREB*2 can reduce the occurrence of ferroptosis [11, 20]. Studies have shown that spinal cord injury in rats involves ferroptosis and is accompanied by an increase in *Ireb2* mRNA [31]. Artemether induces ferroptosis in hepatic stellate cells by inhibiting the degradation of the IREB2 protein, thus playing an important role in antihepatic fibrosis [32]. Knockdown of *IREB*2 can reduce ferroptosis caused by aging-related iron deposition, thereby delaying cell aging and improving neurodegeneration of the auditory cortex [33]. Ferroptosis is related to the increase of IREB2 that occurred in mice intracerebral hemorrhage, and the exosomes from miR-19b-3p-modified adipose-derived stem cells reduced the expression of *Ireb2*, alleviated ferroptosis in intracerebral hemorrhage mice, and improved neurological function [34].

Consequently, we hypothesized that IREB2 plays an important role in ferroptosis of steatotic liver IRI and SHP-HR. Both *in vivo* and *in vitro* experiments showed that the levels of IREB2 mRNA and protein were increased in steatotic liver IRI and SHP-HR. Moreover, the level of the FTH1 protein was decreased and that of TFR1 increased, which led to increased Fe^2+^ levels and, ultimately, ferroptosis. In addition, when we knocked down *Ireb2* mRNA in SHPs, ferroptosis was inhibited significantly, and cell damage was improved in SHP-HR.

More importantly, we found that HO-1/BMMSCs could regulate IREB2 to reduce its protein level, increase the protein level of FTH1, reduce the protein level of TFR1, and finally reduce Fe^2+^, thus inhibiting the occurrence of ferroptosis.

Exosomes are important extracellular vesicles that transmit intercellular information and play an important role in disease treatment [35]. Mesenchymal stem cells have been proven to have a strong ability to produce exosomes. Studies have also shown that the release of exosomes with specific genetic information is an important mechanism by BMMSCs exert their functions [36]. Exosomes have shown great therapeutic potential in liver transplantation-related IRI [37, 38]. BMMSC-derived exosomes have been shown to reduce liver IR injury by regulating the inflammatory response [8]. The exosomes from MSCs induced by human pluripotent stem cells and exosomes from human liver stem cells both have a protective effect on liver IRI [39, 40]. Our previous study found that HO-1/BMMSC-exosomes can reduce the inflammatory injury of intestinal epithelial cells and improve the structure of intercellular tight junction proteins, which is an important mechanism by which HO-1/BMMSCs play a protective role [10]. Therefore, we further studied the regulatory effects of HO-1/BMMSC-exosomes on ferroptosis and IREB2 *in vitro*. The results showed that inhibition of exosome secretion significantly weakened the role of HO-1/BMMSCs in regulating IREB2, inhibiting ferroptosis, and protecting cells. At the same time, HO-1/BMMSC-exosomes exerted a similar to HO-1/BMMSCs. They could regulate the expression of IREB2, reduce the level of Fe^2+^, reduce the occurrence of ferroptosis, and improve cell damage. Therefore, HO-1/BMMSC-exosomes are the mechanistic effectors by which HO-1/BMMSCs inhibit ferroptosis.

Exosomes carry a large number of functional miRNAs, and their special membrane structure can protect their internal RNA from enzymatic degradation. Therefore, the content of exosomal miRNAs is more stable and higher than that in body fluids [35, 36]. miRNAs can bind to partial complementary regions mainly located in the 3′-UTRs of their target mRNA, resulting in mRNA degradation or translation inhibition. In addition, studies have shown that noncoding RNAs, especially microRNAs, play an important role in regulating ferroptosis [41, 42].

Therefore, we speculated that the molecular mechanism by which HO-1/BMMSC-exosomes regulate IREB2 expression might be the exosomal secretion of an *Ireb2*-targeted miRNA and its transfer to SHPs. We screened and predicted miRNAs that might bind to the 3′-UTR region of rat *Ireb2* using TargetScan, miRDB, miRmap, and miRWalk databases, which identified miR-29a-3p, miR-29b-3p, and miR-29c-3p. Previous studies have shown that the mature forms of miR-29 family members (miR-29a, miR-29b, and miR-29c) are highly conserved in humans, mice, and rats. At the same time, miR-29 has a targeted relationship with IREB2 [43–45] and can affect cellular iron homeostasis by regulating IREB2, reducing intracellular iron transport, promoting mitochondrial integrity, and limiting iron overload-mediated neuroaging-related damage. However, antagonizing or knocking down the function of miR-29 affected the life span and survival rate of vertebrates [30]. The miRNA binding site SNP rs1062980 can change the expression of *IREB2* by regulating the binding of miR-29a [46]. In addition, studies have shown that both miR-29a [47, 48] and miR-29b [49, 50] are involved in cerebral IRI, which can be achieved by inhibiting nuclear factor kappa B (NF-*κ*B), NOD-like receptor protein 3 (NLRP3) pathway activation, and the p53-dependent apoptosis pathway to reduce the inflammatory response of cerebral IRI.

Therefore, we detected the levels of these three miRs in HO-1/BMMSCs exosomes. The results showed that the level of miR-29a-3p was the highest in HO-1/BMMSC exosomes, and it increased significantly in HO-1/BMMSCs exosomes or HO-1/BMMSCs in the SHP-HR coculture system. Furthermore, by using an exosome inhibitor, we found that miR-29a-3p in the SHP-HR coculture system mainly came from the exosomes of HO-1/BMMSCs rather being directly secreted from HO-1/BMMSCs. Although the miR-29a-3p mimic did not significantly affect the level of *Ireb2* mRNA, it may inhibit the translation of *Ireb2* mRNA via posttranscriptional regulation, which is consistent with a previous report [30]. Decreased IREB2 protein and Fe^2+^ levels finally inhibited ferroptosis and alleviated SHP-HR cell injury.

To confirm the antiferroptosis and cell protective effect of HO-1/BMMSC exosomes mediated by miR-29a-3p, transfection of an miR-29a-3p inhibitor significantly reduced the level of miR-29a-3p in the coculture system comprising HO-1/BMMSC exosomes and SHPs; eliminated the regulatory effect of HO-1/BMMSCs exosomes on IREB2, FTH1, and TFR1 protein levels; and blocked the protective effect of HO-1/BMMSC exosomes against ferroptosis and cell injury in SHP-HR.

In summary, our study shows that ferroptosis is an important mechanism of steatotic liver IRI, in which IREB2 plays an important role it. In addition, HO-1/BMMSCs can downregulate the protein level of IREB2 and inhibit ferroptosis through miR-29a-3p in its exosomes, thereby protecting steatotic liver IRI and SHP-HR. However, at present, we cannot conclude that this mechanism is a specific mechanism for steatotic liver IRI. Whether it is applicable to other types of liver requires further research, because relevant studies have shown that ferroptosis occurs in a variety of liver diseases and has many molecular targets and regulatory pathways.

## 5. Conclusion

**Figure 9:**
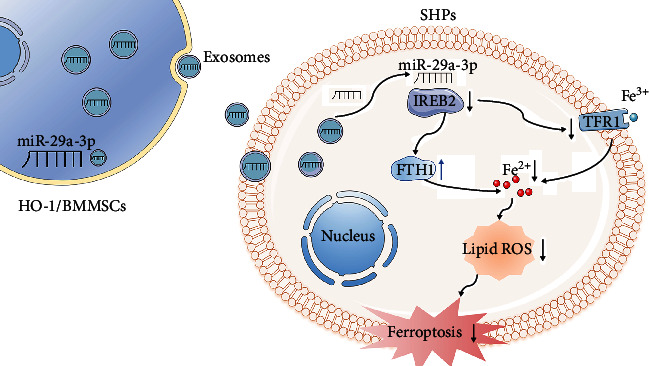
Summary schematic diagram of the mechanisms underlying HO-1/BMMSC-induced ferroptosis modulation to alleviate SHP-HR injury. miR-29a-3p, which targets *IREB*2, is abundant in HO-1/BMMSC-exosomes and could decrease the IREB2 protein level. The reduced IREB2 level led to an increase in the level of FTH1 and decreased the level of TFR1 through posttranscriptional regulation, which ultimately reduced the level of intracellular Fe^2+^ and the production of lipid ROS and inhibited the occurrence of ferroptosis in SHP-HR. BMMSCs: bone marrow mesenchymal stem cells; HO-1: heme oxygenase 1; HR: hypoxia reoxygenation; SHPs: steatotic hepatocytes.

HO-1/BMMSCs can reduce steatotic liver IRI by inhibiting ferroptosis. The molecular mechanism of this inhibition of ferroptosis involves interference with the IREB2/FTH1/TFR1 signal pathway by absorbing HO-1/BMMSC exosomes and transferring miR-29a-3p to steatotic hepatocytes (Figure 9). However, other ferroptosis signaling pathways regulated by HO-1/BMMSCs or HO-1/BMMSC exosomes cannot be completely excluded, which requires further exploration in the future. These observations provide new insights into the therapeutic mechanism of HO-1/BMMSCs and might provide new therapeutic targets and strategies to treat steatotic liver IRI.

## Data Availability

Data supporting the findings of this study are included in the manuscript and the supplementary materials.

## Abbreviations

ALT: Alanine aminotransferase
AST: Aspartate aminotransferase
BMMSCs: Bone marrow mesenchymal stem cells
CD: Cluster of differentiation
CM-DiI: Chloromethyl 1,1′ dioctadecyl-3,3,3′3′-tetramethylindocarbocyanine perchlorate
DAB: Diaminobenzidine
ECD: Expanded criteria donor
ELISA: Enzyme-linked immunosorbent assay
Fer-1: Ferrostatin-1
4-HNE: 4-Hydroxynonenal
FTH1: Ferritin H
GFP: Green fluorescent protein
GPX4: Glutathione peroxidase 4
GSH: Glutathione
HE: Hematoxylin-eosin
HMGB1: High-mobility group box 1
HO-1: Heme oxygenase-1
HRP: Horseradish peroxidase-labeled streptavidin
HR: Hypoxia reoxygenation
IL-1*β*: Interleukin 1 beta
IL-6: Interleukin 6
IREB2: Iron response element-binding protein 2
IRI: Ischemia-reperfusion injury
MDA: Malondialdehyde
miRNA: MicroRNA
mRNA: Messenger RNA
MPO: Myeloperoxidase
NC: Negative control
PBS: Phosphate-buffered saline
PI: Propidium iodide
Ptgs2: Prostaglandin-endoperoxide synthase 2
qRT-PCR: Quantitative real-time reverse transcription PCR
RT1A: RT1 class I, locus A
RT1B: RT1 class I, locus B
SD: Sprague-Dawley
SHPs: Steatotic hepatocytes
Si-RNA: Small interference RNA
TEM: Transmission electron microscopy
TFR1: Transferrin receptor 1
TLR4: Toll-like receptor 4
TNF-*α*: Tumor necrosis factor alpha
TSG101: Tumor susceptibility 101
TUNEL: Terminal deoxynucleotidyl transferase nick-end-labeling
p65: RELA proto-oncogene, NF-*κ*B subunit
p-p65: Phosphorylated p65.

## Supplementary Materials

Supplementary Materials Table S1: sequences for si-RNA. Table S2: sequences for miRNA mimic and inhibitor. Table S3: PCR primer sequences for mRNA. Table S4: PCR primer sequences for microRNA. Figure S1: characteristics of HO-1/BMMSCs *in vitro* and detection of HO-1 expression. Figure S2: establishment of the steatotic liver and IRI model and HO-1/BMMSCs colonization in the liver. Figure S3: the steatotic liver was more susceptible to ferroptosis compared with the normal liver after IRI. Figure S4: inhibition of ferroptosis can reduce steatotic hepatocyte (SHP) hypoxia/reoxygenation (HR) in which IREB2 expression is increased. Figure S5: validation of the knockdown of *Ireb2* mRNA. Figure S6: inhibition of exosomes significantly weakened the role of HO-1/BMMSCs in regulating IREB2, inhibiting ferroptosis, and improving cell viability in the SHP-HR model.
