# Supplementary material for: miR-29a-3p in Exosomes from Heme Oxygenase-1 Modified Bone Marrow Mesenchymal Stem Cells Alleviates Steatotic Liver Ischemia-Reperfusion Injury in Rats by Suppressing Ferroptosis via Iron Responsive Element Binding Protein 2
Source: Oxid Med Cell Longev. 2022 Jun 9;2022:6520789. doi: 10.1155/2022/6520789 (PMC9203237; doi:10.1155/2022/6520789)
Supplement: Supplementary Materials — Table S1: sequences for si-RNA. Table S2: sequences for miRNA mimic and inhibitor. Table S3: PCR primer sequences for mRNA. Table S4: PCR primer sequences for microRNA. Figure S1: characteristics of HO-1/BMMSCs in vitro and detection of HO-1 expression. Figure S2: establishment of the steatotic liver and IRI model and HO-1/BMMSCs colonization in the liver. Figure S3: the steatotic liver was more susceptible to ferroptosis compared with the normal liver after IRI. Figure S4: inhibition of ferroptosis can reduce steatotic hepatocyte (SHP) hypoxia/reoxygenation (HR) in which IREB2 expression is increased. Figure S5: validation of the knockdown of Ireb2 mRNA. Figure S6: inhibition of exosomes significantly weakened the role of HO-1/BMMSCs in regulating IREB2, inhibiting ferroptosis, and improving cell viability in the SHP-HR model. [file 6520789.f1.docx]

**Supplementary Tables**

| **Table S1 Sequences for si-RNA** | |
| --- | --- |
| **Gene (si-RNA)** | **Sequence (5'-3')** |
| si-*Ireb2* (sense) | GCAAUACAGAAUGCACCAATT |
| si-*Ireb2* (anti-sense) | UUGGUGCAUUCUGUAUUGCTT |
| si-*NC* (sense) | UUCUCCGAACGUGUCACGUTT |
| si-*NC* (anti-sense) | ACGUGACACGUUCGGAGAATT |

| **Table S2 Sequences for miRNA mimic and inhibitor** | |
| --- | --- |
| **Gene (miRNA)** | **Sequence (5'-3')** |
| miRNA-29a-3p mimic (sense) | GCAAUACAGAAUGCACCAATT |
| miRNA-29a-3p mimic (anti-sense) | UUGGUGCAUUCUGUAUUGCTT |
| miRNA-29a-3p inhibitor(sense) | UUCUCCGAACGUGUCACGUTT |
| miRNA-29a-3p inhibitor (anti-sense) | ACGUGACACGUUCGGAGAATT |
| miRNA mimic Negative control (sense) | UUCUCCGAACGUGUCACGUTT |
| miRNA mimic Negative control (anti-sense) | ACGUGACACGUUCGGAGAATT |
| miRNA inhibitor Negative control (sense) | CAGUACUUUUGUGUAGUACAA |

| **Table S3 PCR primer sequences for mRNA** | | |
| --- | --- | --- |
| **Gene (mRNA)** | **Primer Sequence (5'-3')** | **Product Size (bp)** |
| *Ho-1*(Forward) | AGGAGATAGAGCGAAACAAGCAGAAC | 150 |
| *Ho-1*(Reverse) | GCTGTGTGGCTGGTGTGTAAGG |  |
| *Ptgs2* (Forward) | CATTTGATTGACAGCCCACCAACTTAC | 112 |
| *Ptgs2* (Reverse) | AGTCATCAGCCACAGGAGGAAGG |  |
| *Ireb2* (Forward) | CATCAGGACAGACGCTCGATG | 159 |
| *Ireb2* (Reverse) | CAGCCAAAACAGCCTTTACACC |  |
| *FTH1* (Forward) | GAACCAGCGAGGTGGACGAATC | 146 |
| *FTH1* (Reverse) | TAGCCAGTTTGTGAAGTTCCAGTAGTG |  |
| *TFR1* (Forward) | GACATTGAGTTGACCCTGGACTATGAG | 132 |
| *TFR1* (Reverse) | TCCACGAGCAGAATACAGCCATTG |  |
| *β-actin* (Forward) | CGCGAGTACAACCTTCTTGC | 200 |
| *β-actin* (Reverse) | ATACCCACCATCACACCCTG |  |

| **Table S4 PCR primer sequences for microRNA** | | | |
| --- | --- | --- | --- |
| **Gene (miRNA)** | **RT-primer** | **Sense primer** | **Anti-sense primer** |
| rno-miR-29a-3p | GTCGTATCCAGTGCAGGGTCCGAGGTGCACTGGATACGACTAACCG | TGCGGTAGCACCATCTGAAATCG | CCAGTGCAGGGTCCGAGGT |
| rno-miR-29b-3p | GTCGTATCCAGTGCAGGGTCCGAGGTGCACTGGATACGACAACACTG | TGCGGTAGCACCATTTGAAATCA |  |
| rno-miR-29c-3p | GTCGTATCCAGTGCAGGGTCCGAGGTGCACTGGATACGACTAACCG | TGCGGTAGCACCATTTGAAATCG |  |
| U6 | TCACGAATTTGCGTGT | CGCTTCGGCAGCACAT | ATTTGCGTGTCATCCTTGC |

**Supplementary Figures**


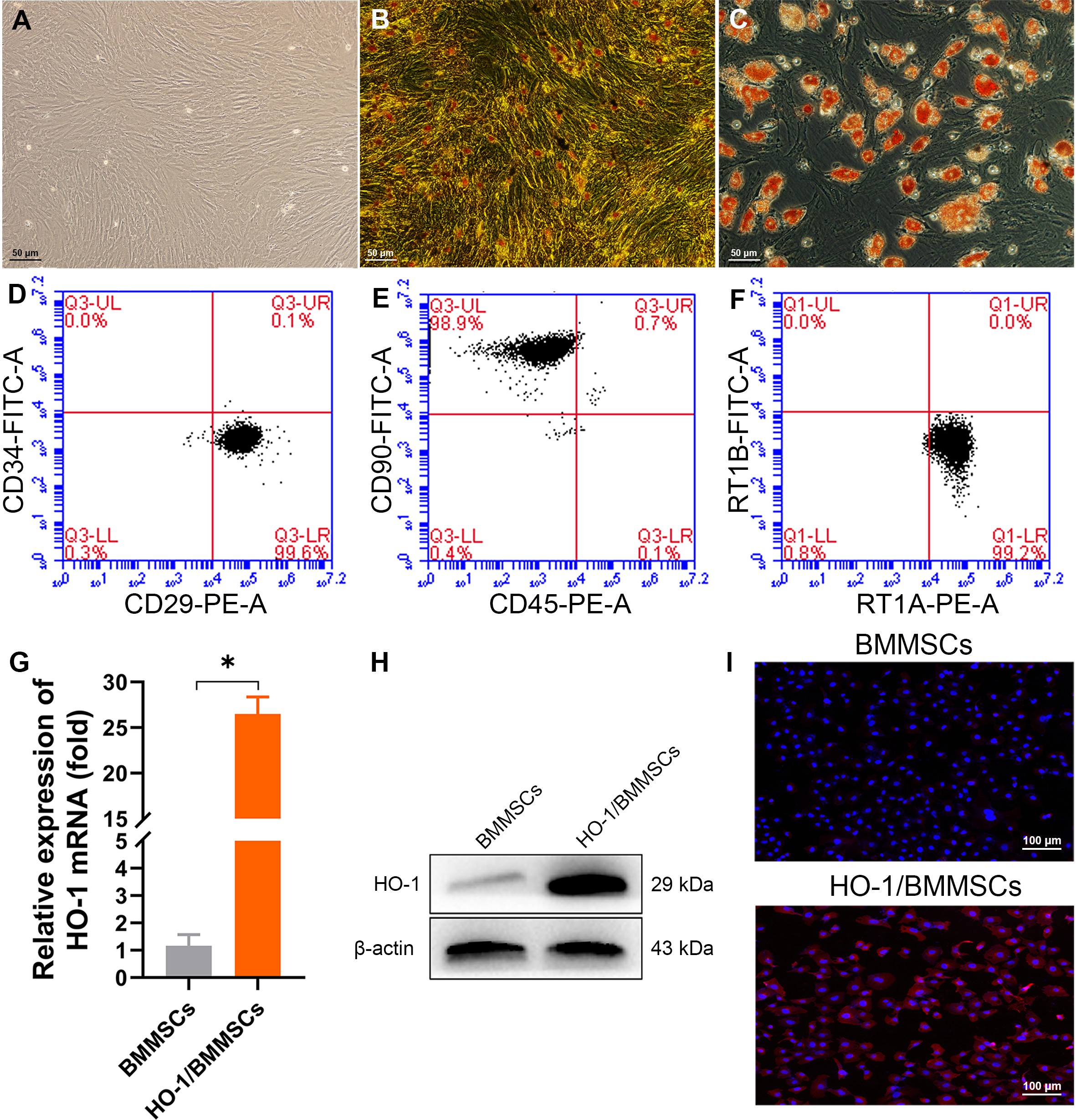


**Fig. S1. Characteristics of HO-1/BMMSCs *in vitro* and detection of HO-1 expression.** (**A**) HO-1/BMMSCs present a typical spindle-shaped appearance of BMMSCs. (**B-C**) HO-1/BMMSCs have the polytropic differentiation potential of adipogenic and osteogenic growth. **(D-F**) HO-1/BMMSCs were positive for CD29,

CD90, and RT1-A and negative for CD34, CD45, and RT1-B, which is same as the basic biological characteristics of BMMSCs. (**G-I**) qRT-PCR, western blotting, and immunofluorescence staining showing higher HO-1 expression of HO-1/BMMSCs than that of BMMSCs. BMMSCs, bone marrow mesenchymal stem cells; HO-1, heme oxygenase 1; CD34, cluster of differentiation 34; CD29, integrin subunit beta 1; CD90, Thy-1 cell surface antigen; CD45, protein tyrosine phosphatase receptor type C; RT1-A, RT1 class I, locus A; RT-1B, RT1 class I, locus B. *n* = 3 per group. ^*^*P <* 0.05.


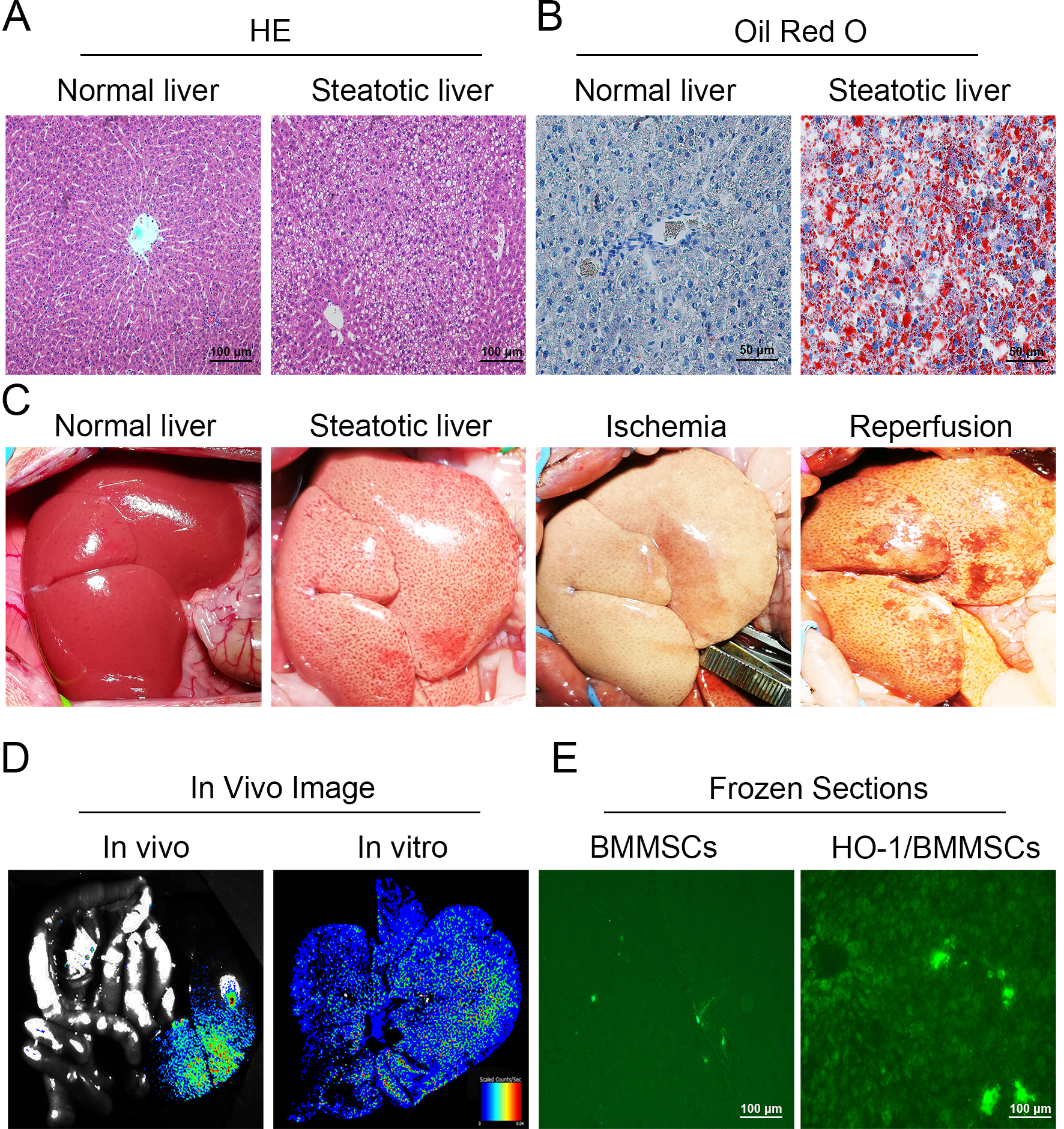


**Fig. S2. Establishment of the steatotic liver and IRI model, and HO-1/BMMSCs colonization in the liver.** (**A**) HE staining of a normal liver and a steatotic liver showed that the steatotic liver had severe (more than 60%) mixed macrovesicular hepatic steatosis. (**B**) Oil Red O staining of a normal liver and a steatotic liver showed that lipid deposition was evident in the steatotic liver with diffuse red particles. (**C**) The normal liver is ruddy with sharp edges, while the steatotic liver is pale yellow and the edges are blunt. Immediate ischemia: the left and middle lobes of the liver become pale because of asphyxia. Immediate reperfusion: liver blood flow recovery with uneven color appearance. (**D**) Animal *in vivo* imaging showing colonization of GFP/HO‑1/BMMSCs in the liver *in vivo* and *in vitro*. (**E**) The number of HO‑1/BMMSCs colonizing the frozen liver sections was greater than that of BMMSCs, indicating that HO-1/BMMSCs prolonged BMMSC survival in vivo. BMMSCs, bone marrow mesenchymal stem cells; HO-1, heme oxygenase 1.


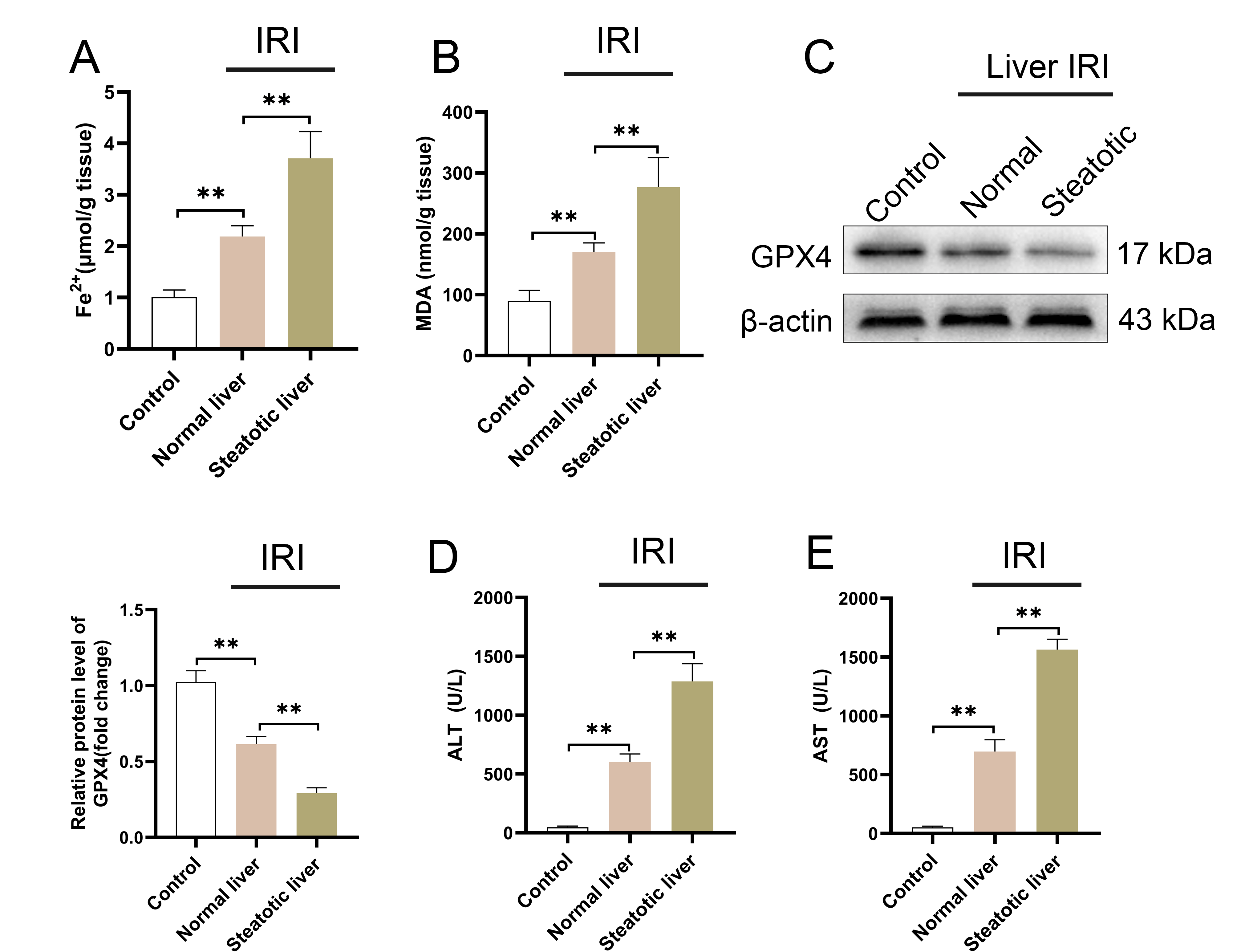


**Fig. S3.** **The steatotic liver was more susceptible to ferroptosis compared with the normal liver after IRI.** (**A**) The Fe^2+^ level detected using colorimetry. (**B**) The MDA content detected using colorimetry. (**C**) GPX4 protein levels assessed by western blotting. (**D-E**) Serum ALT and AST levels after normal and steatotic liver IRI. *n* = 6 per group. Data are presented as the mean ± SEM. ^**^*P <* 0.01.


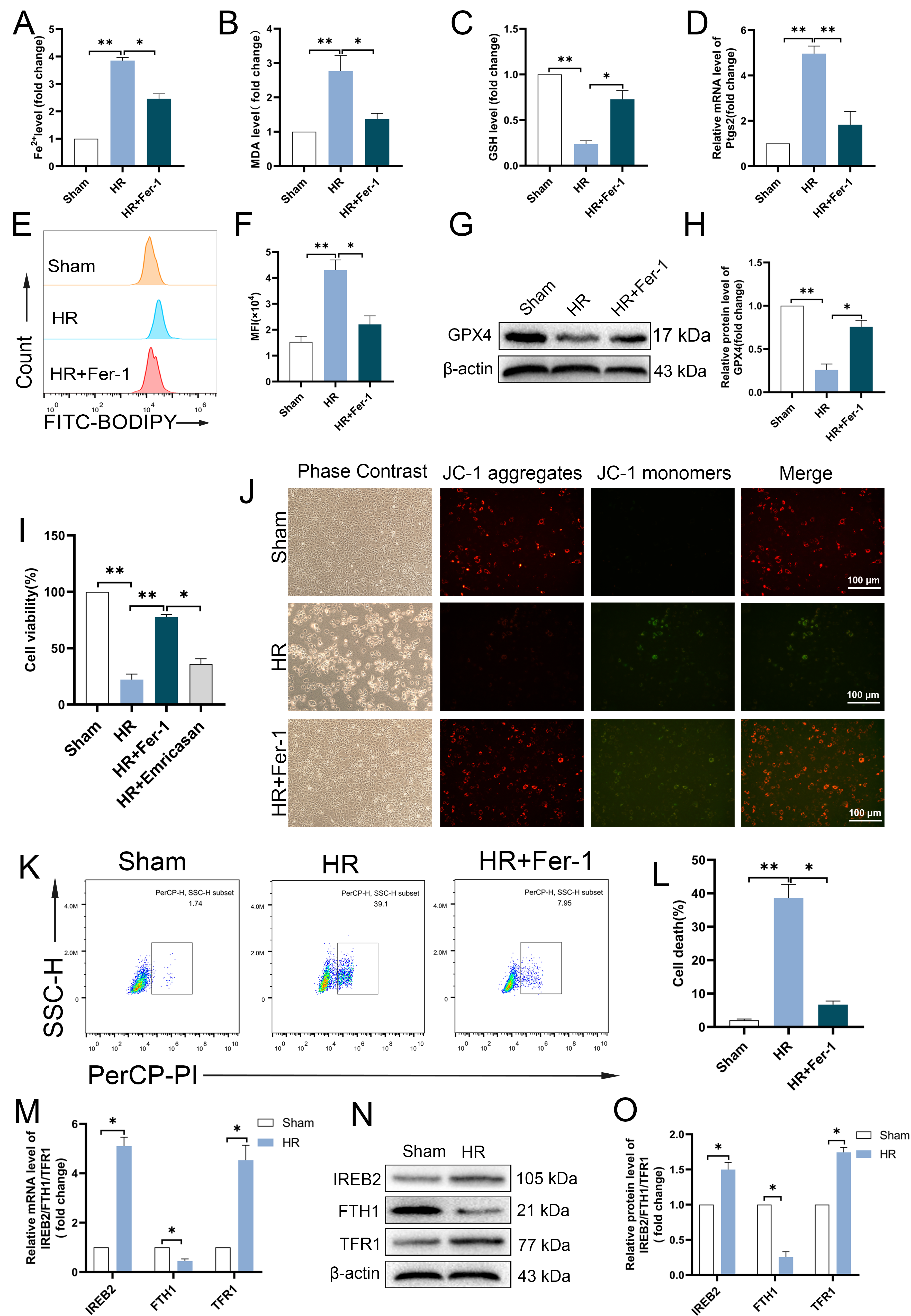


**Fig. S4. Inhibition of ferroptosis can reduce steatotic hepatocyte (SHP) hypoxia/reoxygenation (HR) in which IREB2 expression is increased** Evaluation of ferroptosis and cell injury in the SHP-HR group, with or without Fer-1 treatment. (**A**) The Fe^2+^ level detected using colorimetry. (**B**) The MDA content detected using colorimetry. (**C**) The GSH content detected using colorimetry. (**D**) The *Ptgs2* mRNA level. (**E**) The Lipid ROS level detected using C11-BODIPY and flow cytometry. (**F**) Quantitative analysis of the C11-BODIPY fluorescence intensity. (**G**) GPX4 protein levels, as assessed using by western blotting. (**H**) Semi-quantitative analysis of (**G**). (**I**) Cell viability detected using a CCK-8 kit. (**J**) Cell mitochondrial membrane potential detected using JC-1 staining and fluorescence microscopy, showing that the content of the JC-1 monomer (green) increased and the polymer (red) decreased in HR, representing mitochondrial membrane potential dysfunction. However, HO‑1/BMMSCs reversed this change and improved the mitochondrial membrane potential. (**K**) The cell death ratio detected using PI staining and flow cytometry. (**L**) Quantitative analysis of (**K**). (**M**) *Ireb2*/*Fth1*/*Tfr1* mRNA expression. (**N**) IREB2/FTH1/TFR1 protein levels detected using western blotting. (**O**) Semi‑quantitative analysis of (**N**). *n* = 3 per group. Data are presented as the mean ± SEM. ^*^*P <* 0.05; ^**^*P <* 0.01.


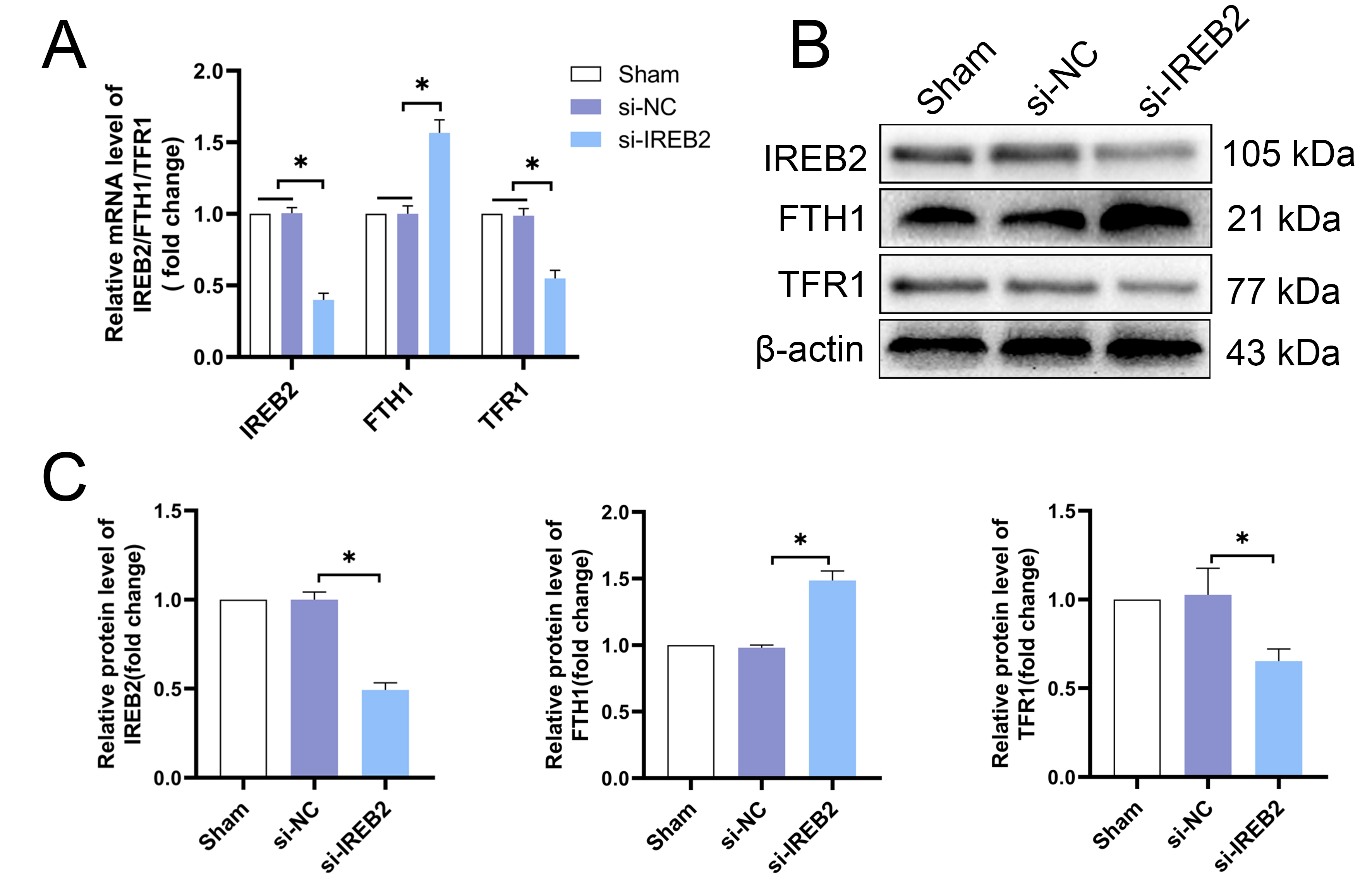


**Fig. S5.** **Validation of the knockdown of *Ireb2* mRNA.** (**A**) si-IREB2 significantly reduced *Ireb2* and *Tfr1* mRNA level in SHPs, but elevated the *Fth1* mRNA level, as compared with those in the Sham and si-NC groups. (**B-C**) si-IREB2 significantly reduced the IREB2 and TFR1 protein levels in SHPs, but elevated the FTH1 protein level, as compared with those in the Sham and si-NC groups. *n* = 3 per group. Data are presented as the mean ± SEM. ^*^*P <* 0.05.


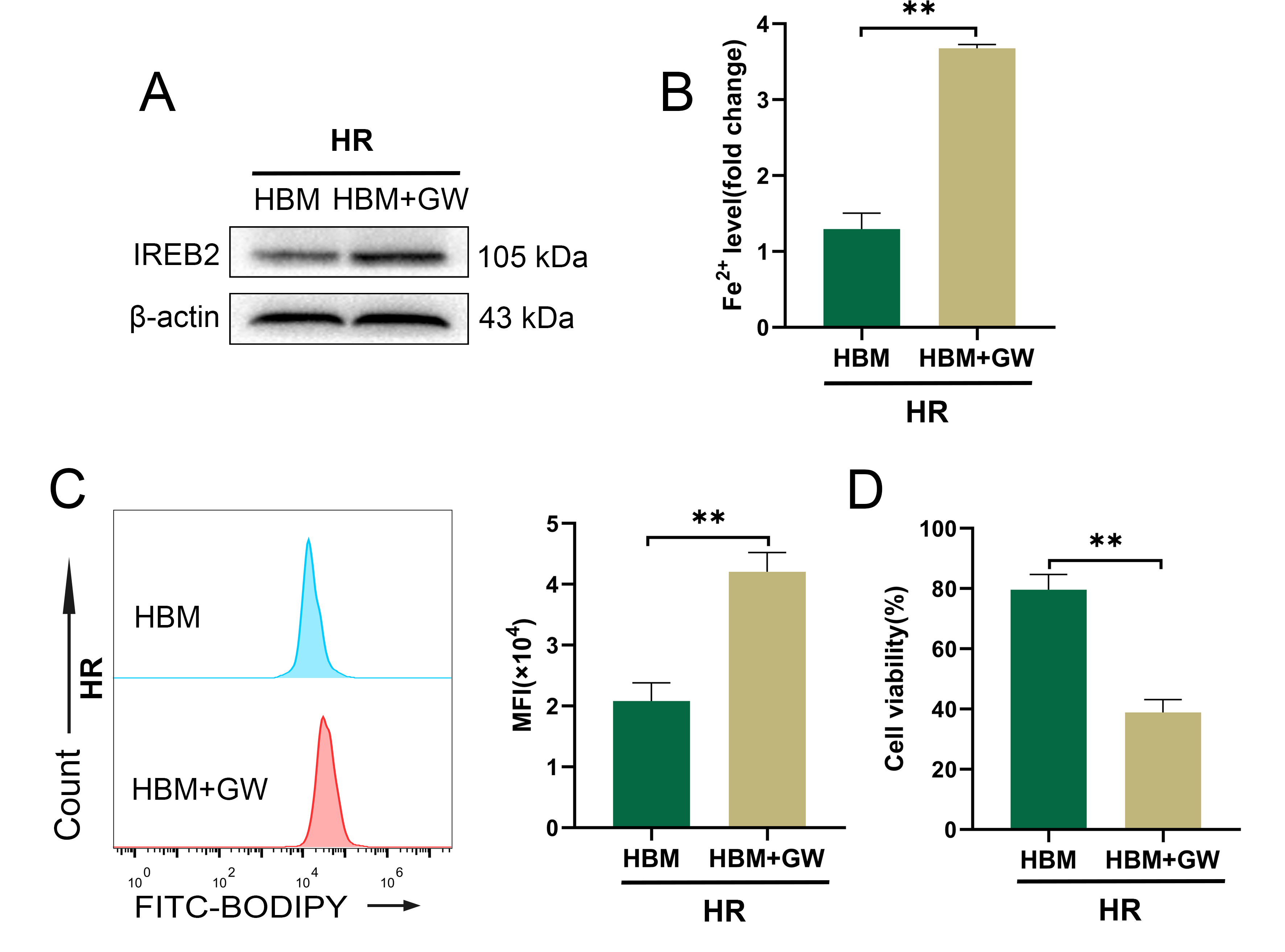


**Fig. S6. Inhibition of exosomes significantly weakened the role of HO-1/BMMSCs in regulating IREB2, inhibiting ferroptosis, and improving cell viability in the SHP-HR model.** (**A**) GW significantly weakened the effect of HO-1/BMMSCs regulation of the IREB2 protein level. (**B**) HO-1/BMMSCs treatment with GW resulted in an increase in the Fe^2+^ level. (**C**) HO-1/BMMSCs treatment with GW resulted in an increase in the Lipid ROS level. (**D**) GW significantly weakened the protect effect of HO-1/BMMSCs in alleviating cell viability. GW; GW4869, an exosome secretion inhibitor; *n* = 3 per group. Data are presented as the mean ± SEM. ^**^*P <* 0.01.
